# Optimal Hypoxia Mimetic Small Molecules for Enhancing Angiogenic Properties of Stem Cells From Human Exfoliated Deciduous Teeth

**DOI:** 10.1111/iej.70191

**Published:** 2026-06-02

**Authors:** Hong Wang, Jiawei Liu, Xintao Yang, Mohamad Koohi‐Moghadam, Shuting Gao, Yuanyuan Han, Dineshi Sewvandi Thalakiriyawa, James Kit Hon Tsoi, Yanqi Yang, Waruna Lakmal Dissanayaka

**Affiliations:** ^1^ Applied Oral Sciences and Community Dental Care, Faculty of Dentistry The University of Hong Kong Hong Kong SAR PR China; ^2^ Department of Diagnostic Radiology, Li Ka Shing Faculty of Medicine The University of Hong Kong Hong Kong SAR PR China; ^3^ Division of Paediatric Dentistry and Orthodontics, Faculty of Dentistry The University of Hong Kong Hong Kong SAR PR China

**Keywords:** angiogenesis, cell survival, HIF‐1α stabilization, hypoxic preconditioning, SHED, small molecules

## Abstract

**Aim:**

Hypoxic preconditioning of cells holds promise for regenerative therapies, yet identifying effective and safe methods for clinical application remains challenging. We aimed to determine optimal hypoxia‐mimetic small molecules (SMs) that stabilize hypoxia‐inducible factor‐1α (HIF‐1α) and their dosages for hypoxic preconditioning in stem cells from human exfoliated deciduous teeth (SHED), and to examine their effects on SHED angiogenic properties.

**Methodology:**

A systematic approach integrating transcriptomics, bioinformatics, and experimental validation was employed. RNA sequencing data from prolyl‐hydroxylase domain 2‐knockdown SHED were queried against the Connectivity Map to identify the top 3 SMs that most closely mimic this hypoxic signature. To establish causality and mechanism, the selective Bcl‐2 inhibitor venetoclax and HIF‐1α siRNA were used. Candidate compounds and conventional agents (CoCl_2_, deferoxamine) were tested for HIF‐1α expression, vascular endothelial growth factor (VEGF) secretion, and cell viability. Pro‐angiogenic functionality was evaluated by in vitro Matrigel and spheroid‐sprouting assays. In vivo angiogenic potential and safety (TUNEL/Ki67) were assessed using a Matrigel plug assay in SCID mice. Additional RNA sequencing compared TW‐37‐ and CoCl_2_‐treated SHED.

**Results:**

CoCl_2_ and deferoxamine increased HIF‐1α and VEGF but showed substantial cytotoxicity at effective doses. TW‐37, ML228, and CPX stabilized HIF‐1α at non‐toxic doses (10 μM, 1 μM, and 7 μM, respectively), and sustained VEGF secretion. SM‐treated SHED‐CM boosted endothelial tube formation and sprouting in vitro, with TW‐37 showing the strongest pro‐angiogenic effect, confirmed to be HIF‐1α‐dependent and separate from Bcl‐2 inhibition. SM‐pretreated‐SHED (24 h) yielded persistent VEGF release and robust in vitro angiogenic activity. In vivo Matrigel plug assays revealed markedly increased vessel density in plugs containing SHED pretreated with TW‐37, ML228, or CPX. In particular, TW‐37‐pretreated SHED showed increased vessel density, enhanced Ki67‐positive proliferation, and no increase in apoptosis (TUNEL). RNA sequencing revealed that TW‐37 upregulated genes associated with angiogenesis, metabolic adaptation, and odontogenesis, indicating a more favourable profile than CoCl_2_.

**Conclusions:**

TW‐37 (10 μM), ML228 (1 μM), and CPX (7 μM) are promising hypoxia‐mimetic regimens in this proof‐of‐concept model. TW‐37 acts through a specific, reversible HIF‐1α‐dependent mechanism, demonstrating a favorable balance between angiogenic enhancement and preliminary safety in regenerative endodontics.

## 1 Introduction

Cell‐based tissue engineering strategies have been widely studied in regenerative medicine, in which neovascularization or angiogenesis is one of the most critical factors that determines the outcome (Kao et al. 2009). Stem cells from human exfoliated deciduous teeth (SHED) have been identified as a population of highly proliferative and clonogenic dental mesenchymal stem cells that are able to induce dentine‐pulp regeneration, angiogenesis, and bone formation (Miura et al. 2003). Because of their superior stem cell properties and the ability to source them through non‐invasive methods from exfoliated teeth, SHED have become a promising cell source in stem cell‐based autologous transplantation and tissue engineering (Miura et al. 2003; Xu et al. 2017). SHED or dental pulp stem cells reside in the pulp tissue surrounded by hard dentine, which allows vasculature only through a narrow opening at the apex of the root canals. Therefore, the oxygen tension at the pulp stem cell niche in vivo is much lower than in the in vitro laboratory culture conditions. However, when the cells are isolated, cultured, and expanded under hyperoxic ambient oxygen levels and then introduced back into the hypoxic in vivo conditions, the sudden deprivation of oxygen will result in poor cell survival and treatment outcomes (Haque et al. 2015; Mas‐Bargues et al. 2019). Following implantation into large tissue defects, only 10%–60% of cells survive, as shown using ischemic models (Shi 2009). The rates could well be lower in cell‐based pulp‐tissue engineering due to the anatomical limitation of the tooth, which receives a limited blood supply (Haque et al. 2015; Mas‐Bargues et al. 2019). Therefore, ensuring adequate cell survival and rapid vascularization following transplantation is a pressing challenge in dental pulp regeneration.

Cells have developed inherent mechanisms to adapt to low oxygen conditions, specifically through the hypoxia‐inducible factor (HIF) family of transcription factors. HIF‐1α is an active subunit of the HIF family, which is a heterodimeric complex containing α and β subunits (HIF‐1α, HIF‐2α, HIF‐3α, and HIF‐β) (Pugh and Ratcliffe 2003). In normoxic conditions, the α subunits of HIFs are hydroxylated at conserved proline residues by prolyl hydroxylase domain enzymes (PHDs), which facilitates recognition by the Von Hippel–Lindau (VHL) E3 ubiquitin ligase complex, leading to ubiquitination and proteasomal degradation of HIF‐1α. Additionally, factor inhibiting HIF (FIH) hydroxylates an asparagine residue in HIF‐α, inhibiting its interaction with transcriptional coactivators and thereby suppressing HIF transcriptional activity without promoting degradation (Hewitson et al. 2002; Ratcliffe 2002). In hypoxia, HIF‐1α is significantly activated, and its degradation is inhibited because hypoxia markedly suppresses the activity of PHD or FIH, leading to the accumulation of HIF‐1α in the nucleus (Hewitson et al. 2002; Hu et al. 2006; Pugh and Ratcliffe 2003; Ratcliffe 2002). Numerous studies by us and others have provided compelling evidence of a strong correlation between HIF‐1α stabilization and angiogenesis, metabolism, erythropoiesis, cell survival, cell proliferation, tumour resistance therapy, and organ protection (Han et al. 2020, 2021, 2022; Linden et al. 2003; Lv et al. 2017; Pan et al. 2021; Shi 2009).

HIF‐1α stabilization or activation induced by hypoxic preconditioning could act as an effective strategy to reduce hypoxia‐induced damage and a potent way to enhance post‐implantation cell survival (Lv et al. 2017; Pan et al. 2021). Interestingly, our previous studies demonstrated that HIF‐1α expression in SHED was significantly stabilized in normoxia following the knockdown of PHD2, which in turn enhanced vascular endothelial growth factor (VEGF) secretion and improved the angio−/vasculogenic properties (Han et al. 2020). Moreover, the in vitro stabilization of HIF‐1α in SHED enhanced post‐implantation cell survival through metabolic reprogramming, inhibiting apoptotic enzymes, and boosted the pulp regeneration in vivo (Han et al. 2022). All those findings established that stabilization of HIF‐1α through PHD2 knockdown markedly augmented the therapeutic potential of SHED by enabling the utilization of a single cell type as the origin for both endothelial and odontogenic lineages. However, the lentiviral‐mediated gene knockdown method that we previously used cannot be applied in the clinical setting due to safety concerns. To successfully address the challenge of translating our strong experimental data into preclinical/clinical applications, we aim to develop safe non‐viral methods. Although hypoxia preconditioning can also be achieved through a hypoxia chamber/incubator (Rinderknecht et al. 2021), the cost and potential air leakage in hypoxia chambers limit their clinical application (Shchaslyvyi et al. 2023; Wenger et al. 2015).

The use of hypoxia‐mimetic chemical agents to inhibit HIF‐1α degradation or enhance HIF‐1α expression has been approved for clinical use, indicating that they can be explored as a safer and clinically applicable strategy for stabilizing HIF‐1α in cell‐based strategies (Corner et al. 2023). The chemical compounds such as cobalt chloride (CoCl_2_) and deferoxamine (DFO) have been examined to activate HIF‐1α expression in various studies (Jiang et al. 2014; Taheem et al. 2018). Accordingly, they can induce the production of VEGF and promote angiogenesis in several types of mesenchymal stem cells (Nowak‐Stȩpniowska et al. 2022). Given the limited experimental data on dental stem cells, these agents have been used empirically in many studies to induce hypoxia‐mimetic conditions in SHED. Our preliminary work suggested that these agents exhibit low efficacy and high cytotoxicity against SHED.

Therefore, in the current study, we aimed to identify optimal chemical HIF stabilizers and characterize appropriate dosage settings in a context‐dependent manner to apply hypoxic preconditioning in SHED effectively. In the current study, using a systematic bioinformatic and scientific experimental approach, we identified three optimal small molecules (SMs) and their dosage that can stabilize HIF‐1α in SHED, promote cell survival, and angiogenic properties both in vitro and in vivo.

## 2 Materials and Methods

The manuscript of this laboratory study has been written following the Preferred Reporting Items for Laboratory studies in Endodontology (PRILE) 2021 guidelines; an overview is provided in Figure S1.

### 2.1 Methodology

This study included in vitro and in vivo experimental approaches.

To assess the efficacy and cytocompatibility of the most commonly used hypoxia mimetic agents, CoCl_2_ and DFO were used at different concentrations to treat SHED and examined the effects on the expression of HIF‐1α and VEGF, as well as cell viability. The effective concentrations demonstrated significant cytotoxicity on SHED.

To systematically identify optimal HIF‐1α‐stabilizing SMs in SHED, RNA sequencing was performed on PHD2 knockdown‐SHED (PHD2KD‐SHED), and the data were processed to identify upregulated and downregulated genes. Using a bioinformatics approach, we compared the transcriptomic profile of PHD2KD‐SHED with the unique signature profile of a drug to create a connectivity map (cMAP) database. Based on the connectivity score and *p*‐value, TW‐37, ML228, and ciclopirox (CPX) were identified as the top three compounds that potentially mimic the transcriptomic signature of PHD2KD‐SHED.

Then, to characterize the effects of the three shortlisted compounds, a series of concentrations was used to treat SHED. The expression of HIF‐1α, the secretion of VEGF, and cell viability at different time points were assessed. To investigate the direct effect of three SMs on the secretion of VEGF, the conditioned medium (CM) was collected and evaluated after SHED were treated with the compounds for 6 h and 24 h (SM‐treated SHED). To further reveal the post‐treatment effect and the possibility of applying hypoxic preconditioning by pretreating with SMs, SHED were first stimulated with the compounds for 24 h, and fresh medium without any compounds was added to continue culturing the cells for another 24 h (SM‐pretreated SHED). Then, the CM was collected to assess VEGF levels. Based on the results, the optimal concentrations of three SMs were identified and applied in the in vitro Matrigel assay and the sprouting assay to determine the functionality of SM‐treated SHED in inducing endothelial vessel‐like structures.

To examine the in vivo angiogenic effects, SHED were pretreated with the three chemicals for 24 h ex vivo and then applied in the in vivo Matrigel plug angiogenesis assay. The Matrigel plugs were harvested at 7 days and examined for the vasculature derived from mouse and human endothelial cells.

To understand the transcriptomic differences of SHED induced by the optimal SM, TW‐37, compared to the most commonly used hypoxia mimetic agent, CoCl_2_, RNA sequencing was performed on SHED treated by these compounds and bioinformatic analysis on the gene expression related to the key biological functions: cell viability, angiogenesis, cell metabolism, and odontogenic differentiation.

As both ML228 and CPX have published evidence confirming their angiogenic effects are HIF‐1α‐dependent (Theriault et al. 2012; Linden et al. 2003), the mechanism of TW‐37—traditionally known as a Bcl‐2 inhibitor—required further validation. To establish causality and ensure the observed effects were not secondary to Bcl‐2 inhibition or off‐target stress, we performed mechanistic studies using the selective Bcl‐2 inhibitor venetoclax and genetic silencing of HIF‐1α via siRNA.

Finally, to further evaluate the translational safety and potential for clinical “hit‐and‐run” preconditioning, we conducted 7‐day washout experiments to monitor the recovery of cell proliferation and sustained pro‐angiogenic activity. Additionally, the in vivo safety of preconditioned SHED was verified through TUNEL (apoptosis) and Ki67 (proliferation) staining within the Matrigel plugs.

### 2.2 Cell Culture and SMs

SHED were purchased from AllCells LLC (Alameda, CA, USA) and characterized for mesenchymal stemness, of which the results were published in our previous study (Han et al. 2022). SHED were cultured in α‐modified Eagle's medium (α‐MEM) supplemented with 10% fetal bovine serum (FBS) and 1% penicillin/streptomycin (P/S) in a 5% CO_2_, 37°C incubator. Human umbilical vein endothelial cells (HUVECs) at passage 1 were purchased from ScienCell (Carlsbad, CA, USA) and cultured in endothelial cell medium (ECM, ScienCell) supplemented with 5% FBS, 1% P/S, and 1% endothelial cell growth supplement at 5% CO_2_, 37°C incubators. SHED from passages 4–7 and HUVECs from passages 3–6 were used in the experiments. To ensure biological reproducibility, all in vitro experiments were conducted in at least three independent biological replicates (*n* = 3). Each biological replicate was derived from biologically distinct samples, utilizing different thaws and passages of the cell line.

CoCl_2_, DFO, and CPX were commercially purchased from Sigma Aldrich (MO, USA) and ML228 from R&D Systems (Minnesota, USA). TW‐37 was purchased from Beyotime (Shanghai, China). Venetoclax was purchased from Selleck (Houston, TX, USA). Based on the literature (Chen et al. 2019; Feng et al. 2020; Jiang et al. 2014; Lifshits et al. 2023; Mori et al. 2021; Müller et al. 2012), concentrations for each SM were used as follows: CoCl_2_ (0 mM, 0.0625 mM, 0.125 mM, 0.25 mM, 0.5 mM, 1 mM), DFO (0 μM, 7.5 μM, 15 μM, 30 μM, 60 μM,120 μM), TW‐37 (0 μM, 1 μM, 2.5 μM, 5 μM, 10 μM, 20 μM), ML228 (0 μM, 0.25 μM, 0.5 μM, 1 μM, 2.5 μM, 5 μM), CPX (0 μM, 1 μM, 2.5 μM, 5 μM, 7 μM, 10 μM), and venetoclax (1 μM).

### 2.3 Western Blot Assay

SHED were treated with different concentrations of the SMs for 6 h and 24 h. The cells were lysed by the RIPA buffer (Thermo Fisher Scientific, CA, USA) with 1% protease & phosphatase inhibitor cocktail (Thermo Fisher Scientific) after being rinsed with PBS three times. The BCA protein detection kit (Thermo Fisher Scientific) was used to detect the protein concentrations. The proteins were separated by sodium dodecyl sulfate‐polyacrylamide gel electrophoresis and transferred onto polyvinylidene difluoride (PVDF) membranes (GE Healthcare Life Science, Little Chalfont, UK). The membranes were blocked with 5% milk in Tris‐buffered saline containing Tween (TBST) at room temperature for 1 h and then immunoblotted at 4°C overnight with the primary antibodies HIF‐1α (BD Biosciences, New Jersey, US), GAPDH (Cell Signalling Technology, MA, USA), Bcl‐2 (Santa Cruz, Dallas, TX, USA), and β‐actin (Santa Cruz). After that, the PVDF membranes were washed with TBST 3 times (5 min each), and the corresponding secondary antibodies (Cell Signalling Technology) were used to incubate the membranes for 1 h at room temperature. After washing with TBST 3 times, the targeted protein signal was developed with the ECL chemiluminescent HRP substrate kit (WesternBrightTM, Advansta, CA, USA), and the membranes were visualized by the detection system (Thermo Fisher Scientific). All the results were quantified by analysing the integrated density with ImageJ software (National Institutes of Health), and the relative quantification of proteins was normalized to GAPDH.

### 2.4 Conditioned Medium

To ensure comparability, SHED in all groups were seeded at identical densities across all groups and cultured to 80% confluence in α‐MEM before treatment with different concentrations of the SMs (CoCl_2_‐treated SHED, DFO‐treated SHED, TW‐37‐treated SHED, ML228‐treated SHED, and CPX‐treated SHED). Conditioned medium (CM) was collected after 6 h (CoCl_2_ 6 h, DFO 6 h, TW‐37 6 h, ML228 6 h, and CPX 6 h) and 24 h (CoCl_2_ 24 h, DFO 24 h, TW‐37 24 h, ML228 24 h, and CPX 24 h). Besides, after SHED was stimulated with TW‐37, ML228, and CPX for 24 h, the supernatant was removed, and fresh medium without any chemicals was added and continued to culture for 24 h (TW‐37‐pretreated SHED, ML228‐pretreated SHED, and CPX‐pretreated SHED). Then the CM was harvested ((TW‐37 24 h) + 24 h, (ML228 24 h) + 24 h, and (CPX 24 h) + 24 h). Internal normalization by equal density was confirmed by assessing cell viability at the time of CM collection using a Live/Dead assay (no significant difference in live cells at collection between groups). All CM was centrifuged at 1000 rpm for 5 min to remove cell debris and was used to detect VEGF expression and in the in vitro Matrigel tube formation assay. Figure 2C outlines the collection of different CM.

### 2.5 Enzyme‐Linked Immunosorbent Assay (ELISA)

The secretory VEGF level in the culture supernatant was assessed by the Human VEGF DuoSet ELISA Kit (R&D Systems, Minnesota, USA) according to the protocol. Briefly, 96‐well microplates were coated with the capture antibody (diluted with PBS, 100 μL per well) overnight and washed 3 times with the Wash Buffer (0.05% Tween 20 in PBS, 400 μL per well). Then the plates were blocked with the Reagent Diluent (1% BSA in PBS, 300 μL per well) for 1 h at room temperature. After washing, the supernatant samples or standards diluted in the Reagent Diluent (100 μL per well) were added to the microplates and incubated for 2 h at room temperature. After repeating the washing step, the Detection Antibody (diluted in the Regent Diluent, 100 μL per well) was added to the plates and incubated for 2 h at room temperature. Then, after washing, the working dilution of the Streptavidin‐HRP (100 μL per well) was added to the plates and incubated for 20 min at room temperature under dark conditions. Subsequently, the plates were washed and incubated with the Substrate Solution (100 μL per well) for 20 min at room temperature without light. Then, the Stop Solution was added to each well (50 μL) and the plates were tapped gently to fully mix. Finally, the absorbance was determined at 450 nm and 540 nm by the microplate reader (SpectraMax M2) and the concentrations of the samples were calculated by the standard curve. The results were expressed as the relative expression of VEGF.

### 2.6 Cell Counting Kit‐8 (CCK‐8) Assay

The cell proliferation was detected using the CCK‐8 reagents (APExBIO, USA) according to the instructions. Briefly, SHED were seeded onto 96‐well plates with 5 × 10^3^ cells and 100 μL medium per well and incubated at 5% CO_2_, 37°C incubators. After 24 h, the cells were treated with different SMs at various concentrations and cultured for 24 h, 48 h, and 72 h, respectively. 10 μL of CCK‐8 solution was added to each well of the plates, and the plates were incubated for 2 h in the incubators. At the end, the absorbance was measured at 450 nm by a microplate reader (SpectraMax M2, Molecular Devices, CA, USA).

### 2.7 Bioinformatic Approach to Identify the Optimal SMs

According to our previous study, RNA sequencing was performed to profile the genome‐wide gene expression profiles of PHD2 knockdown in SHED (Han et al. 2022). The differential expression analysis was conducted to identify the genes that were significantly upregulated and downregulated in PHD2 knockdown cells compared to the control cells. The cMAP tool was utilized to systematically identify the SMs capable of mimicking the transcriptional effects of PHD2 knockdown in SHED. First, the top differentially expressed genes were selected, including both upregulated and downregulated genes with |log Fold Change (FC)| > 1.5. These genes were formatted into a cMAP‐compatible query where upregulated genes were expected to be induced by the candidate compounds and downregulated genes were expected to be suppressed. The gene signature was then compared against the extensive L1000 gene expression database, which contains transcriptional profiles of thousands of drugs applied to human cell lines. Each compound in the database was scored based on the ability to induce a gene expression pattern similar to the PHD2 knockdown signature. Finally, the compounds were ranked based on a connectivity score, which ranged from −100 to +100. High positive scores indicated that the compound induced a gene expression profile similar to PHD2 knockdown, while high negative scores suggested that the compound induced an opposite expression pattern.

### 2.8 Live/Dead Cell Viability Assay

The Live/Dead Assay kit (Thermo Fisher Scientific) was used to detect the cell viability of different compounds on SHED, as per the manufacturer's protocol. After the vials were thawed, 5 μL calcein AM (Component A) and 20 μL ethidium homodimer‐1 (Component B) were added to 10 mL PBS to create a staining solution. Then, the medium was removed from the cells and 100–200 μL of the staining solution was directly added to the cells. The cells were incubated for 30 min at room temperature, and images were captured by the fluorescence microscope (Nikon, Tokyo, Japan). It produced a distinct green colour for live cells and a red colour for dead cells, respectively.

### 2.9 In Vitro Matrigel Assay

In vitro Matrigel tube formation assay was conducted to assess the pro‐angiogenic effects using different CM collected from SHED treated or pretreated with TW‐37, ML228, and CPX. 10 μL of thawed Matrigel matrix (Corning, NY, USA) was added to each well of the μ‐Slide 15‐well plates (Ibidi, Germany) on ice and then the plates were placed in the 37°C incubators for 30 min to induce the gelation. Subsequently, 50 μL of the cell suspension of HUVECs in different CM was seeded onto the wells with Matrigel and observed at regular intervals. Images were taken by an inverted microscope (Nikon) and the nodes were measured by ImageJ software.

### 2.10 In Vitro Sprouting Assay

Sprouting assay was conducted using CM collected from SHED treated with different SMs as described previously (Zhang et al. 2021). Briefly, HUVECs were suspended in ECM containing 20% methylcellulose solution and 5% FBS (8 × 10^4^ cells/mL) and 25 μL per drop of the cell suspension was deposited onto the 100 mm culture plates. The plates were inverted and incubated in the cell culture incubators for 24 h to generate the 3D spheroids. The spheroids were harvested by rinsing the plates with PBS gently and resuspended in the CM. Then the spheroids were added to the solution of the fibrinogen (Sigma Aldrich) and the aprotinin (Sigma Aldrich). After adding the thrombin (Sigma Aldrich) to coagulate, the above spheroid mixture was added to the plates and incubated in the cell culture incubators for 24 h. Images were taken using an inverted microscope (Nikon), and the sprouting length was measured with the ImageJ software.

### 2.11 In Vivo Matrigel Plug Angiogenesis Assay

To evaluate angiogenic potential in vivo, a Matrigel plug assay was performed in severe combined immunodeficient (SCID/CB17) mice (*n* = 6 plugs per group; 12, male, ~6 weeks of age, 20 ~40 g). Animals were purchased through the Laboratory Animal Unit of the University of Hong Kong. Mice were randomly assigned to four groups (control, TW‐37, ML228, and CPX). To minimize inter‐animal variability and comply with the 3Rs principle (Reduction), an incomplete block design was used, in which each mouse received two subcutaneous Matrigel plugs from different experimental groups. The experimental unit for analysis was defined as the individual plug. The animal experimental procedures were approved by the Committee on the Use of Live Animals in Teaching and Research (CULATR, Reference No. 24‐218) of the University of Hong Kong. All animal experiments were performed in accordance with the Guide for the Care and Use of Laboratory Animals published by the US National Institutes of Health and regulations at the Laboratory Animal Unit of the University of Hong Kong.

The in vivo Matrigel plug assay was performed according to the previously established protocol (Yuan et al. 2016). SHED were cultured until reaching 80% confluence in α‐MEM and pretreated with or without the SMs for 24 h. Then, the cells were collected and centrifuged at 1000 rpm for 5 min. The cells were resuspended in ECM and counted to the required number of cells (3 × 10^6^ in 100 μL ECM). 100 μL of cell suspension was mixed with 400 μL of ice‐cold Matrigel (Corning). Mice were anaesthetised with xylazine (10 mg/kg) and ketamine (90 mg/kg) (Alfasan, Woerden, The Netherlands) via intraperitoneal injection. The hair of both lateral hind regions was shaved, and the skin was disinfected with 70% alcohol and a cotton ball. Then, 400 μL of the Matrigel/cell mixture was slowly injected into the subcutaneous space of each side. After injection, the mice were kept in 37°C ICU incubators and transferred to normal cages after recovery. Matrigel plugs were harvested at day 7 after euthanization by overdose of Pentobarbital Sodium (Dorminal 20%, Alfasan, Woerden‐Holland) intraperitoneally. The plugs were rinsed immediately with 1× PBS and fixed with 4% paraformaldehyde for 24 h at room temperature. The whole Matrgiel plug was embedded with paraffin in an orientation that allows the sections to be obtained with the skin and the muscle layers. Then the plugs were vertically sectioned with a 5 μm thickness and stained for histology and immunohistochemistry.

### 2.12 Haematoxylin and Eosin (H&E) Staining

The sections were deparaffinized by passing through 100% xylene twice (each time for 3 min), 100% ethanol twice (each time for 1 min) and rehydrated by immersing in 95% ethanol (1 min), 70% ethanol (1 min) and running tap water (3 min). Sequentially, the sections were immersed in the filtered instant Haematoxylin (Shandon, #6765015) for 3 min and washed with running tap water for 3 min to remove excess stains. Then, the slides were differentiated in 0.1% HCl with 70% ethanol for 1–3 s, and the washing step was repeated. The sections were immersed in the bluing solution (1% ammonia solution) for 1 min and then in running tap water for 3 min. Then the slides were stained in Eosin (0.5% deionized water and 0.05% glacial acetic acid) for 1 min and washed for 10 s. After mounting, the sections were imaged by the inverted microscope (Nikon Eclipse LV100N POL, Tokyo, Japan). The perfused vessels were identified as endothelium‐lined lumens containing red blood cells. Vessel quantification and protein expression were assessed by analysing three non‐adjacent sections per Matrigel plug. For each section, three random fields were imaged at 20× power. For each plug, the counts were averaged to determine the mean vessel density. To assess the relative angiogenic impact of the different treatments, vessel counts in the TW‐37, ML228, and CPX groups were normalized to the mean of the Control group. Data are expressed as a fold‐change relative to the Control. All quantifications were performed by two independent observers blinded to the treatment allocations.

### 2.13 Immunohistochemistry (IHC)

After the deparaffinization and the rehydration processes, the sections were transferred into 1× antigen retrieval solution (Dako, Copenhagen, Denmark) at 95°C for 20 min according to the manufacturer's protocol. After cooling down to room temperature, the slides were washed with PBS (1×) three times (each time for 5 min) and placed into 3% H_2_O_2_ in PBS (15 min) to block the endogenous peroxidase activity. Then, after the washing step, 5% BSA was used as the blocking buffer and added to the samples to incubate for 1 h at room temperature. The slides were incubated with the primary antibody (rabbit anti‐human CD31, Abcam, Cambridge, UK; rabbit anti‐mouse CD31, Abcam; Ki67, Proteintech China, Wuhan, China) diluted with the blocking buffer in a humidity box at 4°C overnight. The slides were repeated with the washing step and incubated with the secondary antibody according to the manufacturer's protocol (Mouse and Rabbit Specific HRP/DAB Detection IHC kit, Abcam). Briefly, the slides were incubated with the biotinylated goat anti‐polyvalent for 10 min at room temperature and washed four times. Then, the streptavidin peroxidase was applied to incubate the sections for 10 min at room temperature. After the washing step, the DAB solution (30 μL DAB chromogen in 1.5 mL DAB substrate) was added to the sections for 10 s–10 min. The slides were rinsed to remove the excess DAB and observed under the inverted microscope. Subsequently, the sections were stained with Haematoxylin for counterstain and washed with water. The slides were dehydrated and mounted after drying. The imaging was achieved by the microscope (Nikon Eclipse LV100N POL). The human and mouse CD31‐positive areas were measured in 3 randomly selected regions per section from 3 samples per group using ImageJ, and the percentage area was calculated over the total area.

### 2.14 Quantitative Real‐Time PCR

Quantitative real‐time polymerase chain reaction (RT‐PCR) was performed to detect the mRNA expression levels of the target genes—HK1, HK2, and LDHA. Total RNA was extracted by using an RNAfast200 kit (Fastagen) according to the manufacturer's instructions, and the NanoDrop ND‐1000 spectrophotometer (NanoDrop Technologies) was used to detect RNA concentrations and qualities. The PCR analysis was carried out with the TB Green Premix kit (Takara) according to the protocol, and the relative quantification of gene expression was analysed by the 2^−ΔΔCt^ method. The primer sequences used are as follows: β‐actin (F: CATTGCTGACAGGATGCAGA, R: CTGCTGG AAGGTGGACAGTGA), HK1 (F: CTGCTGGTGAAAATC CGTAGTGG, R: GTCCAAGAAGTCAGAGATGCAGG), HK2 (F: GCTCAAGACAAGGGGCATCT, R: GTCGTCACAGGTGC TCTCAA), LDHA (F: ATGGCAACTCTAAAGGATCAGC, R: CCAACCCCAACAACTGTAATCT).

### 2.15 HIF‐1α Knockdown by siRNA

The silencing of HIF‐1α in SHED was accomplished through the transfection of pre‐prepared siRNA (siHIF‐α: 4390824, negative control: 4390843, Thermo Fisher Scientific) with Lipofectamine RNAiMAX reagents (Invitrogen) according to the manufacturer's protocol. siRNA carrying a non‐significant sequence was used as a negative control. siRNA‐lipid complexes were added into the cells and after 24 h, TW‐37 was then added into the cells to continue to culture for 24 h. The cells were collected for western blot and PCR analysis and the culture medium was collected to detect the secretion levels of VEGF by ELISA.

### 2.16 Bioinformatic Analysis of SM‐Treated SHED

To understand the transcriptomic alterations of TW‐37‐induced SHED with a previously recognized hypoxia mimetic agent (CoCl_2_) at the transcriptomic levels, RNA sequencing between TW‐37‐induced SHED and CoCl_2_‐induced SHED was performed. SHED was treated with 10 μM TW‐37 or 0.5 mM CoCl_2_ for 3 h and 24 h, respectively (CoCl_2_‐3 h group and TW‐37‐3 h group, CoCl_2_‐24 h group and TW‐37‐24 h group, 3 biological replicates per group). The samples were collected using Trizol reagent (Thermo Fisher Scientific) according to the manufacturer's protocol and sent to Biomarker Technologies Corporation (Beijing, China) for RNA sequencing. The purity, concentration, and integrity of the RNA samples were assessed using NanoDrop, Qubit 2.0, and Agilent 2100. Total high‐quality RNAs with a 260/280 absorbance ratio of 1.8–2.0 were utilized for library construction and sequencing. The mapping clean reads were aligned using HISAT2. A transcriptome assembly and gene expression measurement guided by a reference genome were conducted using StringTie. To appropriately show the expression level of each transcript, the quantity of mapped reads was normalized by the length of the transcripts. Fragments per kilobase of transcript per million fragments mapped (FPKM) was utilized to quantify the expression level of a gene or transcript by StringTie employing the maximum flow technique. More than 93.37% of bases in each sample had a *Q*‐score no less than Q30 (Table S1). The differential expression analysis was conducted by DESeq2 to identify the genes that were significantly upregulated and downregulated in TW‐37‐treated cells compared with the cells treated with CoCl_2_. The criteria for differentially expressed genes were established as |log FC| ≥ 1.5 and false discovery rate (FDR) < 0.01. The data have been deposited in a public OMIX repository (Open Archive for Miscellaneous Data, https://ngdc.cncb.ac.cn/omix/) under the accession ID: OMIX016024. A list of genes related to hypoxia, angiogenesis, glycolysis, odontogenesis, oxidative phosphorylation, and cell survival was summarized from the GeneCards database (Table S2). Single‐sample gene set enrichment analysis (ssGSEA) was performed against each gene set and a total of 6 ssGSEA enrichment scores for each sample were obtained at 24 h. Pathway enrichment analyses using the Gene Ontology (GO) and Kyoto Encyclopedia of Genes and Genomes (KEGG) at 3 h were performed based on the upregulated genes and the downregulated genes.

### 2.17 TUNEL Assay

TUNEL assay was conducted with the In Situ Cell Death Detection Kit (Roche, Switzerland) according to the manufacturer's protocol. Briefly, after deparaffinization and hydration, the sections were treated with a cell‐permeabilization solution. The TUNEL reaction mixture was added to the samples, and the sections were incubated in a dark wet box. Then the samples were rinsed with PBS three times, and the converter‐peroxidase was applied. After the washing step, DAB substrate was added to the sections, and the washing step was repeated. The imaging was performed using a digital scanner (Pannoramic MIDI, 3DHISTECH) by the Shiyanjia Lab (Wuhan, China). The percentage of TUNEL‐positive cells was measured with ImageJ software.

### 2.18 Statistical Analysis

All in vitro experiments were conducted in three independent biological replicates (*n* = 3). Each biological replicate was derived from biologically distinct samples, utilizing different thaws and passages of the cell line. Within each independent experiment, technical replicates (triplicate wells) were included to verify assay consistency. The values from these technical replicates were averaged to generate a single, representative data point for each biological replicate. All quantitative results and subsequent statistical analyses are based strictly on these independent biological replicates. The data were presented as mean ± standard deviation (SD). All statistical assumptions, including normality and equal variance, were verified before parametric testing. Statistical analysis was carried out by Student's *t*‐test to compare two groups, one‐way analysis of variance (ANOVA) with Tukey's post‐test, or two‐way ANOVA in multiple comparisons using the GraphPad Prism software (San Diego, CA), with *p* < 0.05 being considered statistically significant.

#### 2.18.1 In Vivo Matrigel Plug Assay

Statistical analysis was performed using GraphPad Prism 11. To account for the nested experimental design (two Matrigel plugs per mouse), a Linear Mixed‐effects Model (LMM) was employed. In this model, the Treatment Group was treated as a fixed effect, while the Mouse ID was included as a random effect to account for within‐subject correlation. Post hoc comparisons between groups were performed using Tukey's multiple comparison test. For all analyses, a *p*‐value < 0.05 was considered statistically significant.

## 3 Results

### 3.1 CoCl_2_ and DFO Induced HIF‐1α Stabilization, Promoted the Secretion of VEGF, but Significantly Inhibited Cell Viability in SHED

**FIGURE 1:**
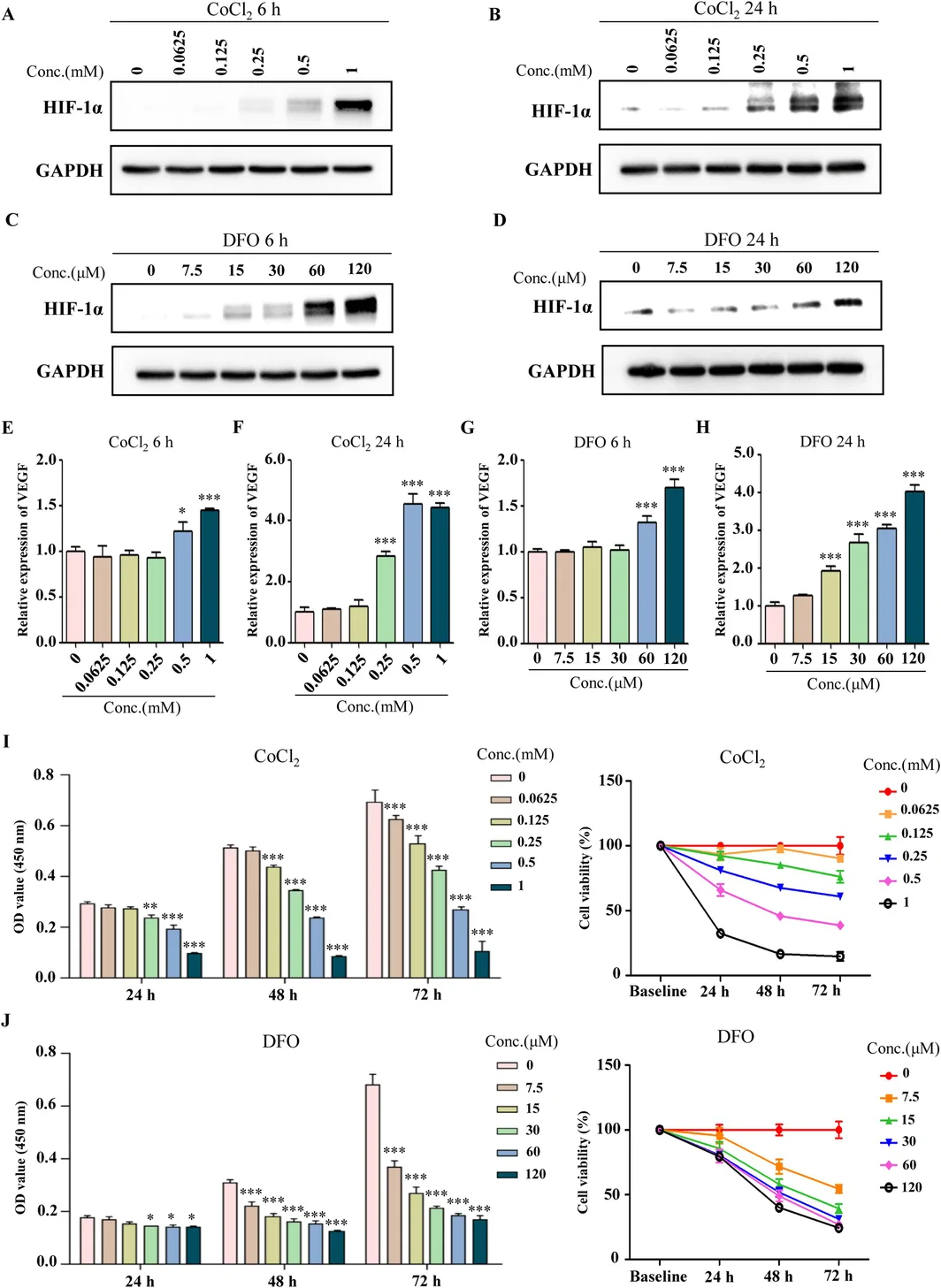
CoCl_2_ and DFO induced HIF‐1α stabilization, promoted VEGF secretion, but significantly inhibited cell viability in SHED. HIF‐1α expression in SHED treated with various concentrations of CoCl_2_ for 6 h (A) and 24 h (B), and DFO for 6 h (C) and 24 h (D), as shown by western blotting. Relative secretory VEGF levels of SHED treated with various concentrations of CoCl_2_ for 6 h (E) and 24 h (F), and DFO for 6 h (G) and 24 h (H), as shown by ELISA. OD values and percentage cell viability indicating cell viability of SHED treated with various concentrations of CoCl_2_ (I) and DFO (J) at 24 h, 48 h, and 72 h, as shown by the CCK‐8 assay. *n* = 3, *p < 0.05, **p < 0.01, and ***p < 0.001.

To assess the efficacy of commonly used hypoxia mimetic agents on SHED, CoCl_2_ and DFO were used to treat SHED at a series of concentrations and examined for the HIF‐1α expression, VEGF secretion, and cell viability. As shown by the western blotting, CoCl_2_ (Figure 1A,B) and DFO (Figure 1C,D) increased the expression of HIF‐1α proteins at both 6 h (Figure 1A,C) and 24 h (Figure 1B,D) in SHED in a dose‐dependent manner. Concurrently, the ELISA assay showed that both CoCl_2_ (Figure 1E,F) and DFO (Figure 1G,H) could promote the secretion of VEGF. There was a significant increase in VEGF when the concentrations of CoCl_2_ were above 0.5 mM at 6 h (Figure 1E) and 0.25 mM at 24 h (Figure 1F). Meanwhile, DFO increased the expression of VEGF at concentrations of 60 μM and 120 μM after 6 h of treatment (Figure 1G), and after 24 h, DFO significantly upregulated VEGF proteins starting from a concentration of 15 μM (Figure 1H).

However, CoCl_2_ (Figure 1I) and DFO (Figure 1J) exhibited significant cytotoxicity on SHED, inhibiting cell viability as shown by the CCK‐8 assay. After 24 h of treatment, CoCl_2_ greatly reduced cell viability starting at a concentration of 0.25 mM, while DFO also reduced cell viability starting at 30 μM. Both agents significantly suppressed cell viability from this point onward. Almost all concentrations (except 0.0625 mM CoCl_2_ at 48 h) of both compounds showed substantial cytotoxicity to the cells after 48 and 72 h of treatment.

Based on the results of the ELISA and CCK‐8 assays at 24 h, nearly all the effective concentrations of the two compounds that could increase the secretory protein levels of VEGF showed significant inhibition of SHED cell viability. For example, the effective concentrations of CoCl_2_ that increased VEGF were 0.25 mM, 0.5 mM, and 1 mM, but they suppressed the cell viability of SHED. These findings suggest that CoCl_2_ and DFO act as hypoxia‐mimetic agents with low efficacy and high cytotoxicity in SHED.

### 3.2 TW‐37, ML228, and CPX, as Optimal HIF‐1α Stabilizers, Effectively Stabilized HIF‐1α in SHED and Promoted VEGF Secretion

**FIGURE 2:**
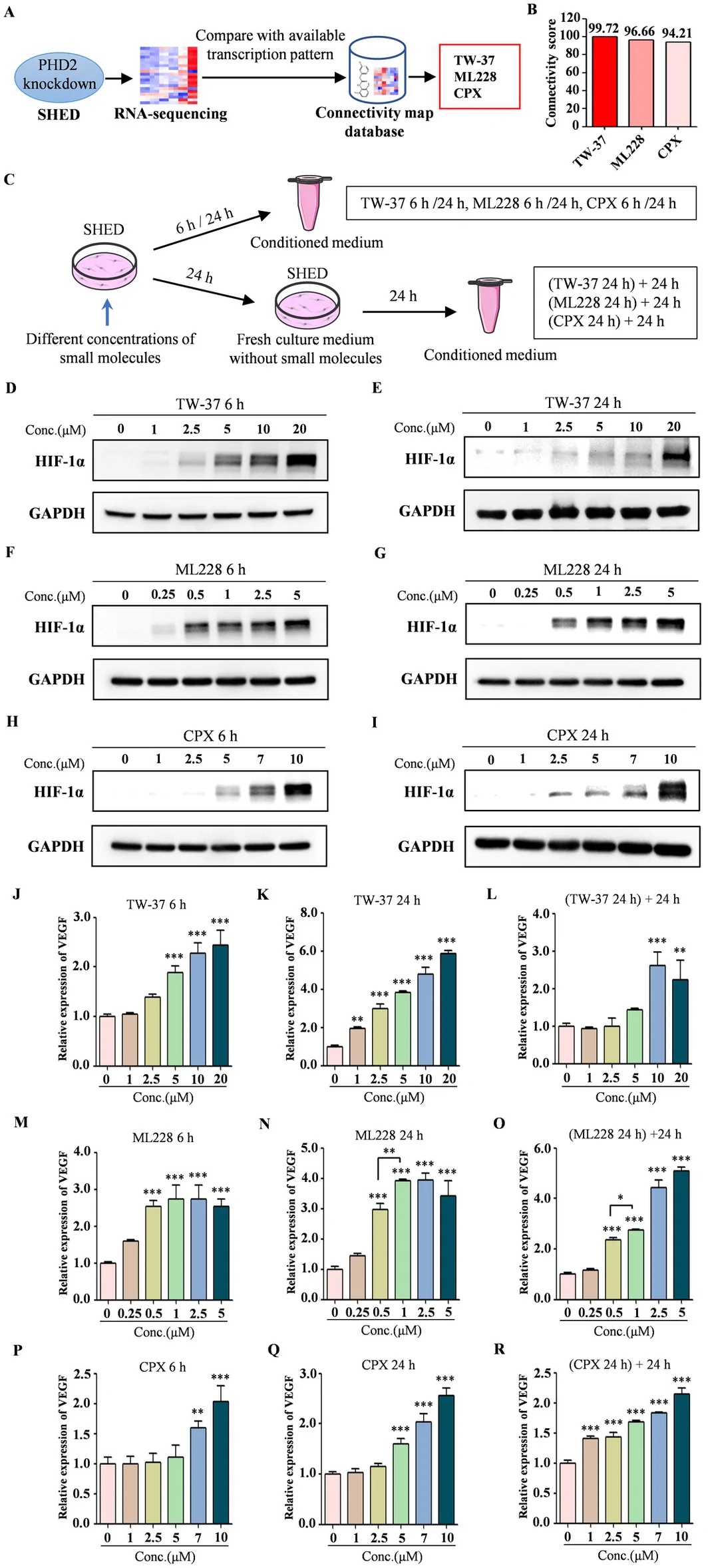
TW‐37, ML228, and CPX induced HIF‐1α stabilization and promoted the secretion of VEGF in SHED. (A) Schematic illustration of the bioinformatic approach of screening of the hypoxia‐mimetic SMs. (B) The top three SMs with their connectivity scores of the bioinformatics analysis: TW‐37 (99.72), ML228 (96.66), and CPX (94.21). (C) Schematic illustration of the collection of CM under different treatment conditions and the labelling of each CM. HIF‐1α expression in SHED treated with different concentrations of TW‐37 at 6 h (D) and 24 h (E), ML228‐treated SHED at 6 h (F) and 24 h (G), and CPX‐treated SHED at 6 h (H) and 24 h (I) as shown by western blotting. (J–R) Secretory VEGF levels in different CM outlined in (C) assessed by ELISA: TW‐37 6 h (J)/24 h (K), and (TW‐37 24 h) + 24 h (L), ML228 6 h (M)/24 h (N), and (ML228 24 h) + 24 h (O), CPX 6 h (P)/24 h (Q), and (CPX 24 h) + 24 h (R). *n* = 3, * *p* < 0.05, **p < 0.01, and ***p < 0.001.

The top three SMs that potentially mimic the transcriptomic signature of PHD2KD‐SHED were identified by comparing the transcriptomic profile of PHD2KD‐SHED with the unique signature profile of a drug on a connectivity map (cMAP) database (Figure 2A,B). The highest scoring compound was TW‐37, with a connectivity score of 99.72, followed by ML228 (96.66) and CPX (94.21) (Figure 2B). Then, to assess the effects of the three SMs on the expression of HIF‐1α and VEGF, SHED were incubated with varying concentrations of different SMs for 6 h and 24 h (Figure 2C). The western blot assay showed that the protein expression levels of HIF‐1α were significantly increased by TW‐37 in SHED under normoxia in a dose‐dependent manner at 6 h (Figure 2D) and 24 h (Figure 2E), respectively. In non‐treated SHED and in SHED treated with doses lower than 0.25 μM of TW‐37, almost no protein of HIF‐1α was detected. At concentrations 0.25 μM or higher, TW‐37 induced upregulated levels of HIF‐1α, with the maximum levels observed at the highest concentration tested, 20 μM, for both 6 h and 24 h. A similar phenomenon of HIF‐1α stabilization was also detected in ML228 (Figure 2F,G) and CPX‐treated SHED (Figure 2H,I). Under the treatment for 6 h and 24 h, ML228 and CPX induced HIF‐1α expression from 0.5 μM (ML228) and 2.5 μM (CPX) concentrations, with the highest at the concentrations of 5 μM (ML228) and 10 μM (CPX). These results revealed that TW‐37, ML‐228, and CPX could function as hypoxia‐mimetic agents that stabilize HIF‐1α in SHED.

Based on our previous study on the role of HIF‐1α–VEGF pathway in the angiogenic property of SHED (Han et al. 2020; Han et al. 2021), the expression levels of VEGF were detected. The ELISA results showed that TW‐37 significantly upregulated VEGF expression in SHED at 5 μM at 6 h (Figure 2J) and 1 μM at 24 h (Figure 2K). Meanwhile, ML228 (from 0.5 μM at 6 h and 24 h) (Figure 2M,N) and CPX (from 7 μM at 6 h and 5 μM at 24 h) (Figure 2P,Q) also significantly increased the VEGF levels in SHED.

Moreover, we were interested in examining VEGF secretion levels after removing direct SM stimulation, as this is the condition most likely to be applied clinically. The results demonstrated that SHED treated with 10 μM and 20 μM for 24 h secreted significantly higher amounts of VEGF even after 24 h of changing into the fresh medium [(TW‐37 24 h) + 24 h] compared to the control group, with 10 μM TW‐37‐pretreated SHED showing the highest expression level (Figure 2L). Similarly, after removing ML228 or CPX, the pretreated SHED still secreted a higher level of VEGF at 24 h, with the maximum levels observed at the concentrations of 5 μM in the [(ML228 24 h) + 24 h] group (Figure 2O) and 10 μM in the [(CPX 24 h) + 24 h] group (Figure 2R). Taken together, the results suggested that TW‐37, ML228, and CPX could significantly enhance the secretion of VEGF in a dose‐dependent way in SHED while in direct contact as well as 24 h after pre‐treatment.

**FIGURE 3:**
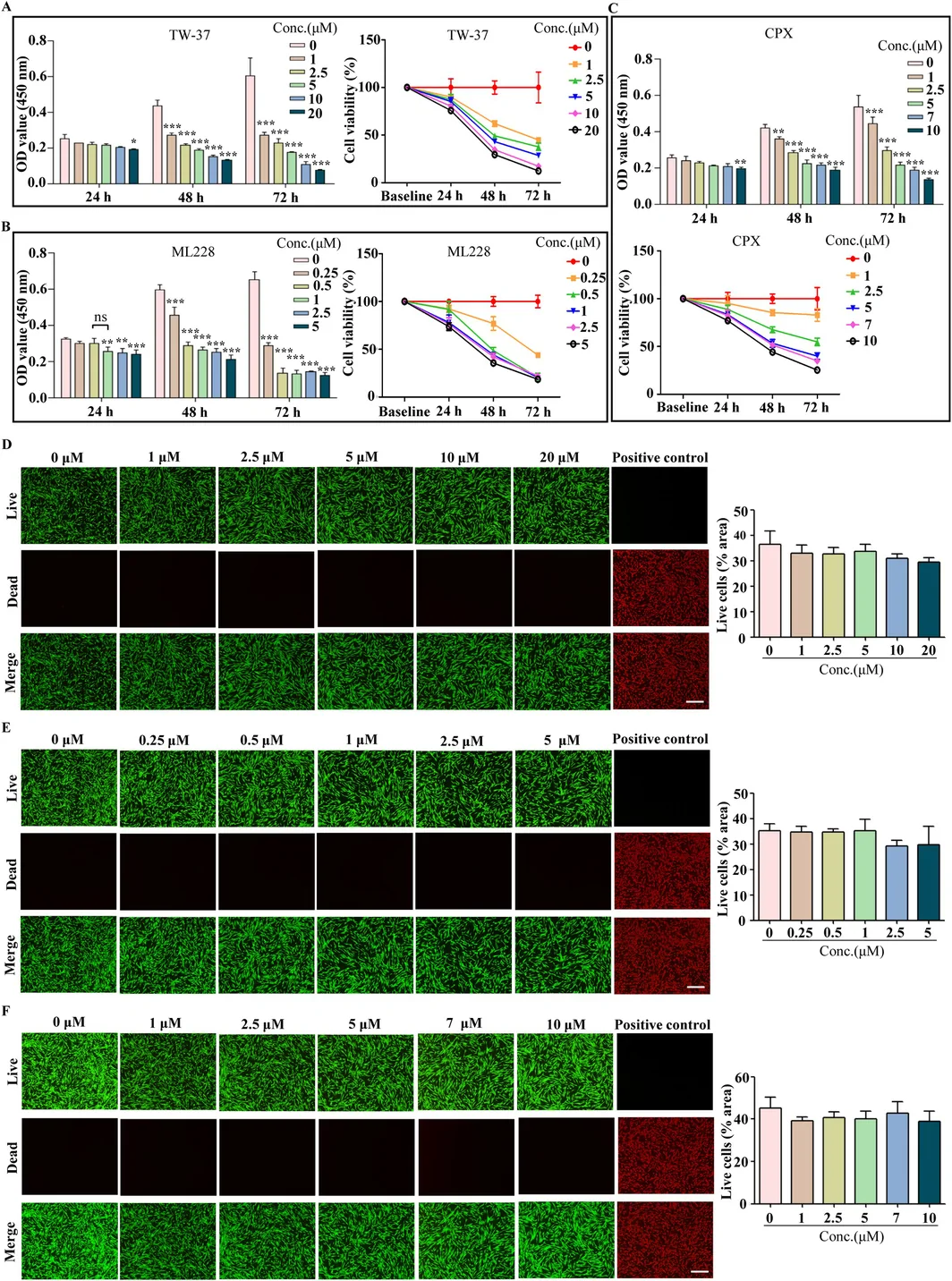
Effects of TW‐37, ML228, and CPX on the viability of SHED. OD values and percentage cell viability of SHED treated with different concentrations of (A) TW‐37, (B) ML228, (C) CPX at 24 h, 48 h, and 72 h. D. Representative images and quantification of the Live/dead assay for SHED treated with different concentrations of (D) TW‐37, (E) ML228, and (F) CPX at 24 h. Green fluorescence: Live cells, Red fluorescence: Dead cells. Positive control: SHED treated with 70% ethanol as a reference for dead cells (far right column). Scale bar = 500 μm. *n* = 3, *p < 0.05, **p < 0.01, and ***p < 0.001.

**FIGURE 4:**
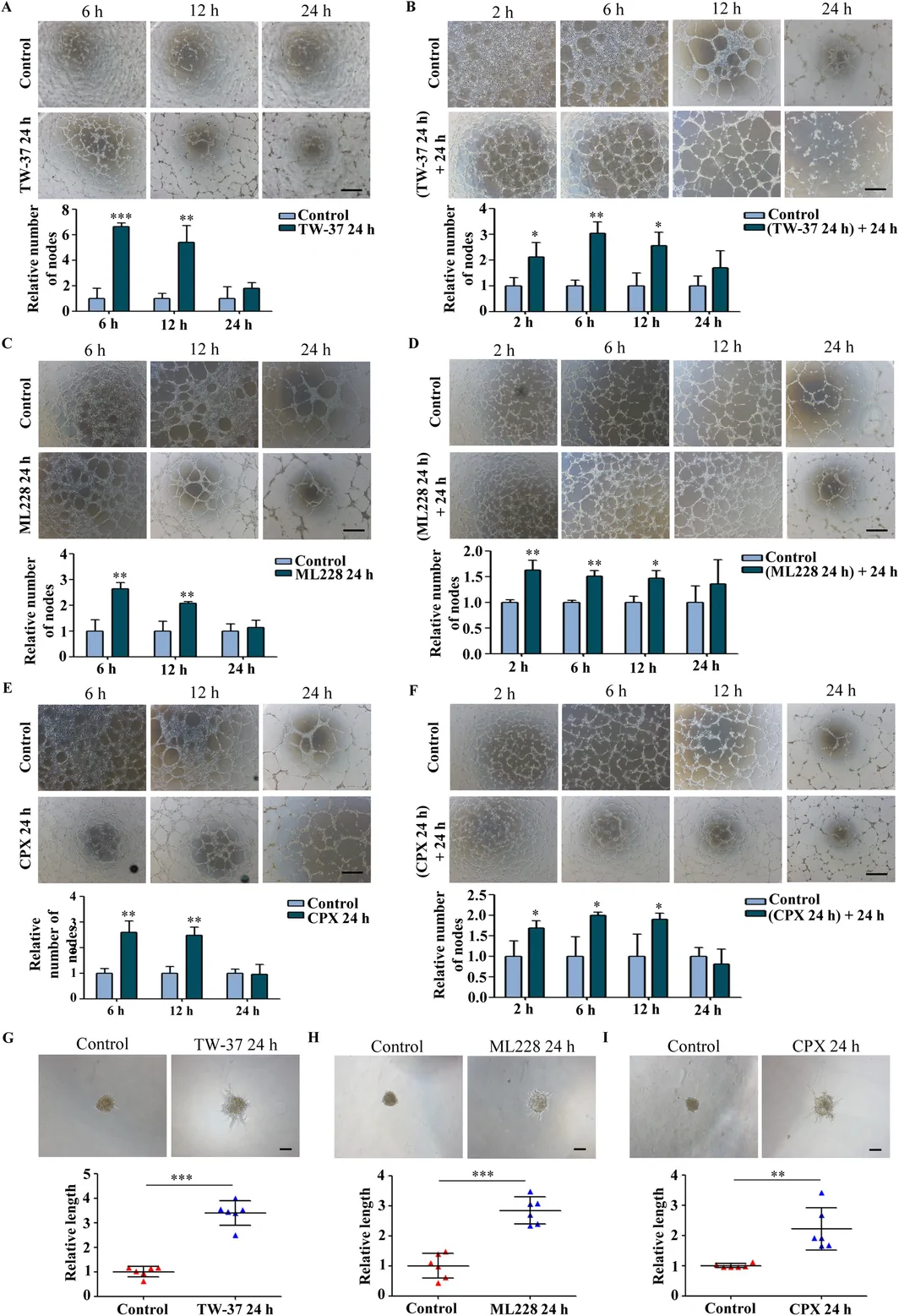
SHED treated with TW‐37, ML228, and CPX enhanced vascular tube formation and sprouting by HUVECs. Representative images and quantified vascular nodes of in vitro Matrigel assay with HUVECs in CM from (A) TW‐37‐treated SHED at 6 h, 12 h, and 24 h; (B) TW‐37‐pretreated SHED at 2 h, 6 h, 12 h, and 24 h; (C) ML228‐treated SHED at 6 h, 12 h, and 24 h. (D) ML228‐pretreated SHED at 2 h, 6 h, 12 h, and 24 h; (E) CPX‐treated SHED at 6 h, 12 h, and 24 h. (F) CPX‐pretreated SHED at 2 h, 6 h, 12 h, and 24 h. Representative images and quantification of endothelial sprouts of the sprouting assay by HUVECs in CM from (G) TW‐37‐treated SHED, (H) ML228‐treated SHED, and (I) CPX‐treated SHED at 24 h. Control: CM from untreated SHED. TW‐37 24 h, ML228 24 h, CPX 24 h: CM from SHED treated by TW‐37 (10 μM), ML228 (1 μM), or CPX (7 μM) for 24 h, respectively. (TW‐37 24 h) + 24 h, (ML228 24 h) + 24 h, (CPX 24 h) + 24 h: SHED was pretreated with TW‐37 (10 μM), ML228 (1 μM), or CPX (7 μM) for 24 h and after the supernatant was removed, SHED was continued to be cultured in fresh medium without any compounds for 24 h, then the new CM was collected, respectively. (A–F), Scale bar = 500 μm. (G–I), Scale bar = 100 μm. *n* = 3, *p < 0.05, **p < 0.01, and ***p < 0.001.

Moreover, the three SMs induced varying levels of VEGF secretion in SHED. Following treatment with 10 μM of TW‐37, the secretion levels of VEGF in SHED were more than 2‐fold higher at 6 h (Figure 2J) and 4‐fold higher at 24 h (Figure 2K) compared to their respective baseline of non‐treated cells. Meanwhile, with the concentration of 1 μM, ML228 significantly upregulated VEGF expression by more than 2‐fold at 6 h (Figure 2M) and 3‐fold at 24 h (Figure 2N) compared to the untreated control groups. In comparison, CPX induced only about a 1.6‐fold increase at 6 h (Figure 2P) and a 2‐fold increase at 24 h (Figure 2Q) in VEGF levels at the concentration of 7 μM. When the three compounds were respectively used to pretreat SHED for 24 h and then removed to allow SHED to be cultured without any SMs for 24 h, 10 μM TW‐37 (Figure 2L) and 1 μM ML228 (Figure 2O) still elevated VEGF expression by over 2.6‐fold, whilst 7 μM CPX (Figure 2R) only resulted in a 1.8‐fold rise. Although ML228 pretreatment at 2.5 μM and 5 μM (Figure 2O) and CPX at 10 μM (Figure 2R) increased VEGF levels after 24 h, these concentrations were cytotoxic to SHED (Figure 3).

### 3.3 TW‐37, ML228, and CPX Were Not Cytotoxic on SHED at 24 h

At 24 h of treatment, TW‐37, except at its highest concentration of 20 μM, did not significantly inhibit the cell viability of SHED (Figure 3A), while ML228 only showed significant inhibition of cell viability at 1, 2.5, and 5 μM (Figure 3B), and CPX at 10 μM (Figure 3C), as determined by CCK‐8 assay. At 48 h and 72 h, almost all concentrations of the three SMs exhibited significant cytotoxicity to SHED (Figure 3A–C).

Interestingly, the results of the live/dead assay demonstrated that almost no distinct dead cells were observed at 24 h when comparing the TW‐37‐treated SHED with the untreated SHED, indicating that TW‐37 inhibited SHED proliferation but did not induce cell death (Figure 3D). Similar results were also observed on ML228 (Figure 3E) and CPX (Figure 3F). However, when extending the cell culture time to 48 h and 72 h, high concentrations of TW‐37 above 2.5 μM (Figure S2A,B), ML228 above 0.5 μM (Figure S2C,D), and CPX at 10 μM (Figure S2E,F) led to an increase in the number of dead cells. Moreover, cell morphology changed, with cells becoming elongated following treatment with the small‐molecule SMs (Figures S2 and S3). All these results revealed that TW‐37, ML228, and CPX inhibited cell viability without inducing cell death during short‐term stimulation (24 h), whereas they induced cell death and caused morphological alterations following prolonged stimulation. However, given the 24 h pretreatment duration and the effective concentrations required for HIF‐1α stabilization and VEGF enhancement, 10 μM TW‐37, 1 μM ML228, and 7 μM CPX were identified as safe pretreatment doses.

### 3.4 SHED Treated With TW‐37, ML228, and CPX Enhanced Vascularization by HUVECs

Based on the above results, 10 μM TW‐37, 1 μM ML228, and 7 μM CPX were identified as optimal concentrations for subsequent experiments. To examine the angiogenic effects of SMs, an in vitro Matrigel assay of vascular tube formation by HUVECs was conducted using the CM collected from SHED cultures treated with each SM for 24 h. Significantly higher numbers of vascular nodes were observed in HUVECs treated with the CM from TW‐37‐ (Figure 4A), ML228‐ (Figure 4C), or CPX‐ (Figure 4E) treated SHED than those in the CM from the untreated SHED at 6 h and 12 h. The relative number of nodes was more than 5 times higher in the TW‐37 24 h group than in the control group, whereas it was less than 3 times higher in the ML228 24 h group and the CPX 24 h group than in their respective control groups at 6 h and 12 h.

Then, to examine the indirect effect of the compounds on the tube formation, CM from the SM‐pretreated SHED was used in the assay. The results showed that HUVECs in the [(TW‐37 24 h) + 24 h] group (Figure 4B), [(ML228 24 h) + 24 h] group (Figure 4D), and [(CPX 24 h) + 24 h] group (Figure 4F) gave rise to an extensive well‐connected vascular tube network with an increased number of nodes compared with control HUVECs at 2 h, 6 h, and 12 h. HUVECs in the [(TW‐37 24 h) + 24 h] group formed more nodes by more than 2‐fold compared to those in the control group, while the [(ML228 24 h) + 24 h] group and [(CPX 24 h) + 24 h] group showed less than a 2‐fold increase.

Additionally, the in vitro fibrin sprouting assay showed that the spheroids in the CM from SHED treated with TW‐37 (Figure 4G), ML228 (Figure 4H), or CPX (Figure 4I) had significantly longer sprouts than those in the control groups at 24 h after embedding. The spheroids in the TW‐37 24 h group demonstrated a higher sprouting length with a more than 3‐fold increase than the control spheroids, while there was less than a 3‐fold increase in the ML228 24 h group and the CPX 24 h group. These data suggested that SHED induced by the three hypoxia mimetic agents could enhance vessel formation.

### 3.5 Hypoxic Preconditioning With TW‐37, ML228, and CPX Pre‐Treatment Boosted the Angio−/Vasculogenesis of SHED In Vivo

**FIGURE 5:**
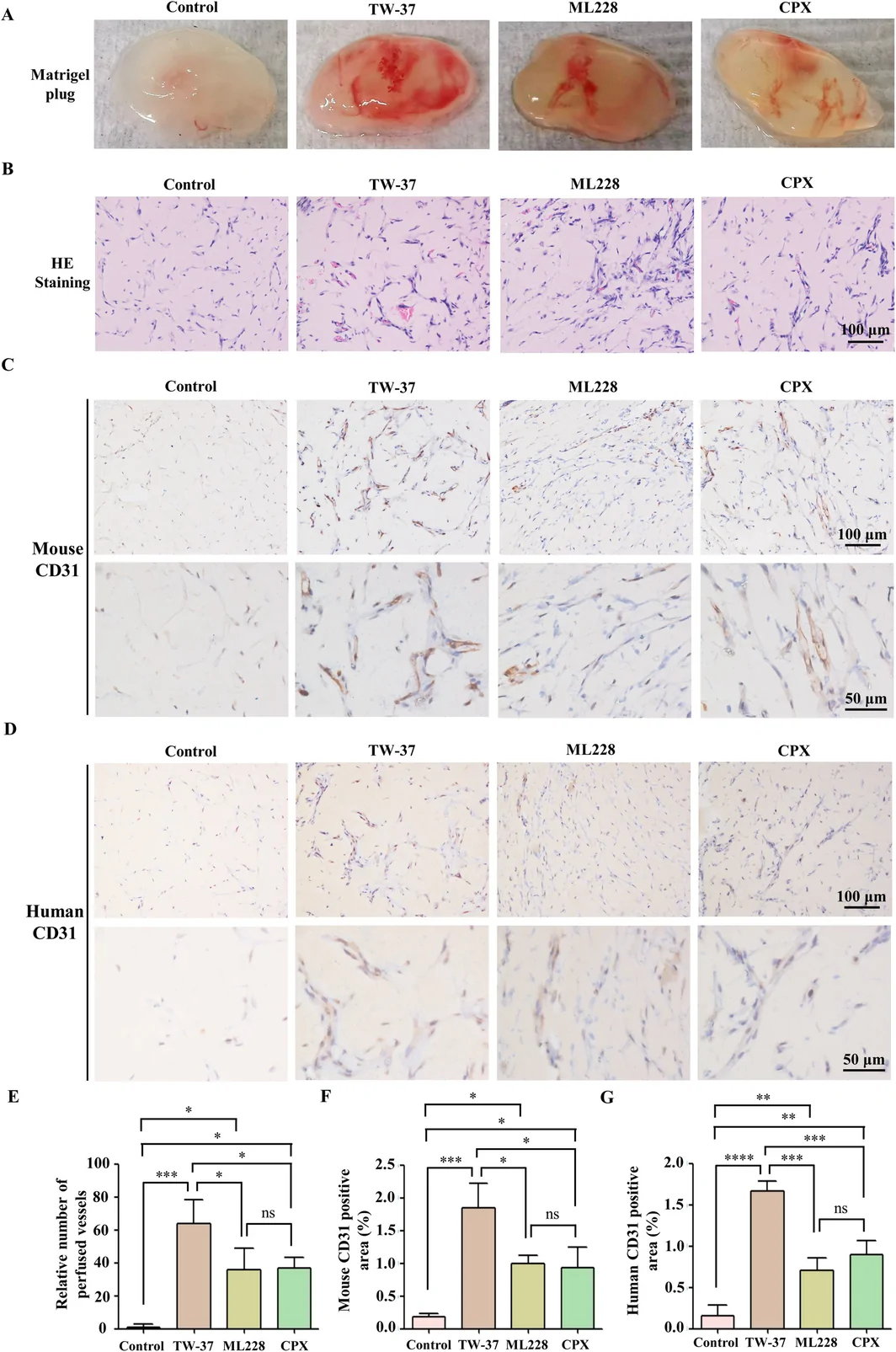
Hypoxic preconditioning with TW‐37, ML228, and CPX boosted the angio−/vasculogenesis of SHED in vivo. (A) Representative macroscopic images of the Matrigel plugs retrieved after 7 days of implantation. Representative micrographs of (B) haematoxylin and eosin (H&E) stained sections, (C) immunohistochemistry for mouse CD31, and (D) immunohistochemistry for human CD31. Quantification of (E) number of perfused vessels, (F) area of mouse CD31 positivity, and (G) area of human CD31 positivity. *n* = 6, *p < 0.05, **p < 0.01, ***p < 0.001, and ****p < 0.0001.

As shown in the in vivo Matrigel plug assay, SHED pretreated with the SMs (10 μM TW‐37, 1 μM ML228, and 7 μM CPX) for 24 h in vitro showed a significant increase in the vascularization after the in vivo implantation for 7 days (Figure 5). To examine the formation of blood vessels within the Matrigel plugs, H&E staining was conducted. The results showed that TW‐37‐, ML228‐, or CPX‐pretreated SHED had a higher number of perfused vessels compared with the untreated SHED (Figure 5B,E). More perfused vessels were formed in SHED with the pretreatment of TW‐37 compared to the ML228 and CPX groups, and there was no significant difference between the ML228 group and the CPX group. Immunohistochemical staining revealed a significantly higher number of mouse CD31‐positive vessels and cells in SHED following preconditioning with hypoxia mimetic agents compared to the control groups (Figure 5C,F). Additionally, in the pretreated SHED groups, a higher number of cells expressed the human CD31 protein compared with the control groups (Figure 5D,G). Interestingly, SHED in the TW‐37 group had more mouse and human CD31 expression compared to those in the ML228 and CPX groups. These findings indicated that hypoxic preconditioning with TW‐37, ML228, and CPX promoted the endothelial differentiation of SHED in vivo and enhanced the capability to recruit host endothelial cells and vascular ingrowth into the implants. TW‐37 exhibited a more substantial pro‐angiogenic effect than ML228 and CPX on SHED by higher expression levels of mouse and human CD31 and the number of blood vessels formed in the plugs after transplantation.

### 3.6 Transcriptomic Alterations in TW‐37‐Treated SHED Compared With CoCl_2_ ‐Treated SHED

**FIGURE 6:**
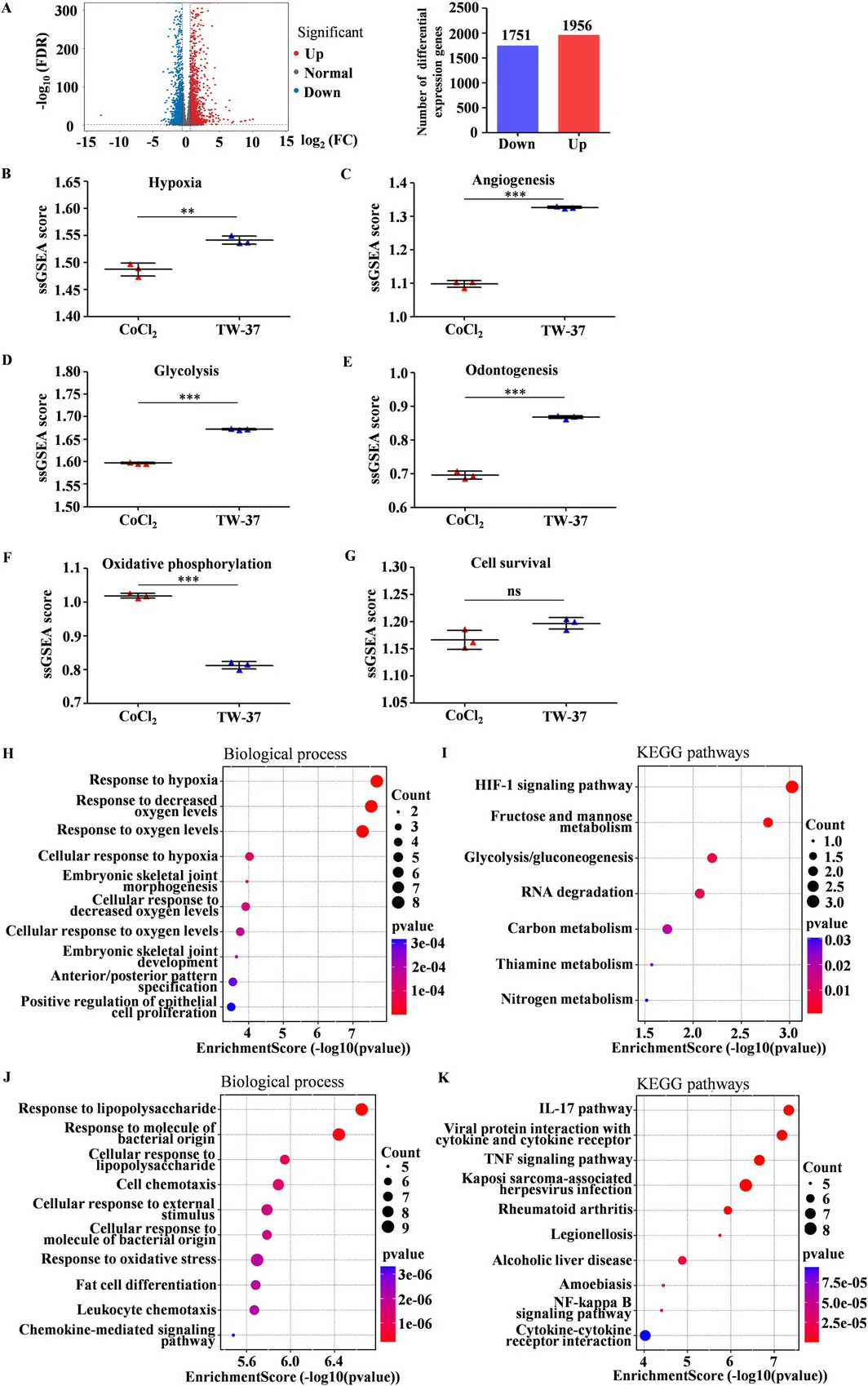
Transcriptomic alterations in TW‐37‐treated SHED compared with CoCl_2_‐treated SHED. (A) Number of differentially downregulated and upregulated genes at 24 h. (B–G) ssGSEA specific for hypoxia (B), angiogenesis (C), glycolysis (D), odontogenesis (E), oxidative phosphorylation (F), and cell survival (G). (H) Enrichment of the biological process of GO analysis by the upregulated genes. (I) KEGG pathway enrichment by the upregulated genes. (J) Enrichment of the biological process of GO analysis by the downregulated genes. (K) KEGG pathway enrichment by the downregulated genes. *n* = 3, **p < 0.01 and ***p < 0.001.

Analysis of differential expression genes (DEGs) identified a total of 3707 genes that were differentially expressed between the SHED groups treated with TW‐37 and CoCl_2_, including 1956 upregulated genes and 1751 downregulated genes when the cells were cultured for 24 h (Figure 6A). Then, to further gain insights into the differential functions in TW‐37‐treated SHED compared with CoCl_2_‐treated SHED, analysis of ssGSEA, KEGG, and GO pathway enrichments was performed. The ssGSEA scores indicated that the enrichment degree of TW‐37‐treated SHED was significantly greater than that of CoCl_2_‐treated SHED at 24 h, in relation to the processes of hypoxia (Figure 6B), angiogenesis (Figure 6C), glycolysis (Figure 6D), and odontogenesis (Figure 6E). The results indicated that TW‐37‐treated SHED could exhibit stronger angiogenic ability and metabolic adaptation than CoCl_2_‐treated SHED. Accordingly, the oxidative phosphorylation was lower in the TW‐37 group compared to the CoCl_2_ group (Figure 6F), indicating a stronger glycolytic shift in TW‐37‐treated SHED compared to CoCl_2_ treatment. Regarding cell survival‐related genes, although the TW‐37‐treated SHED group had a higher score, there was no statistical significance between the two groups (Figure 6G). Biological process of GO (Figure 6H) analysis and KEGG analysis (Figure 6I) from the upregulated genes revealed that TW‐37 specifically up‐regulated those genes that play important roles in response to hypoxia and the HIF‐1 signalling pathway. Meanwhile, the pathways analysis from the downregulated genes (Figure 6J,K) suggested that TW‐37 down‐regulated those genes that functioned in response to lipopolysaccharide and the IL‐17 pathway.

### 3.7 TW‐37‐Mediated Angiogenic Potential Is HIF‐1α‐Dependent and Distinct From Its Bcl‐2 Inhibitory Activity

**FIGURE 7:**
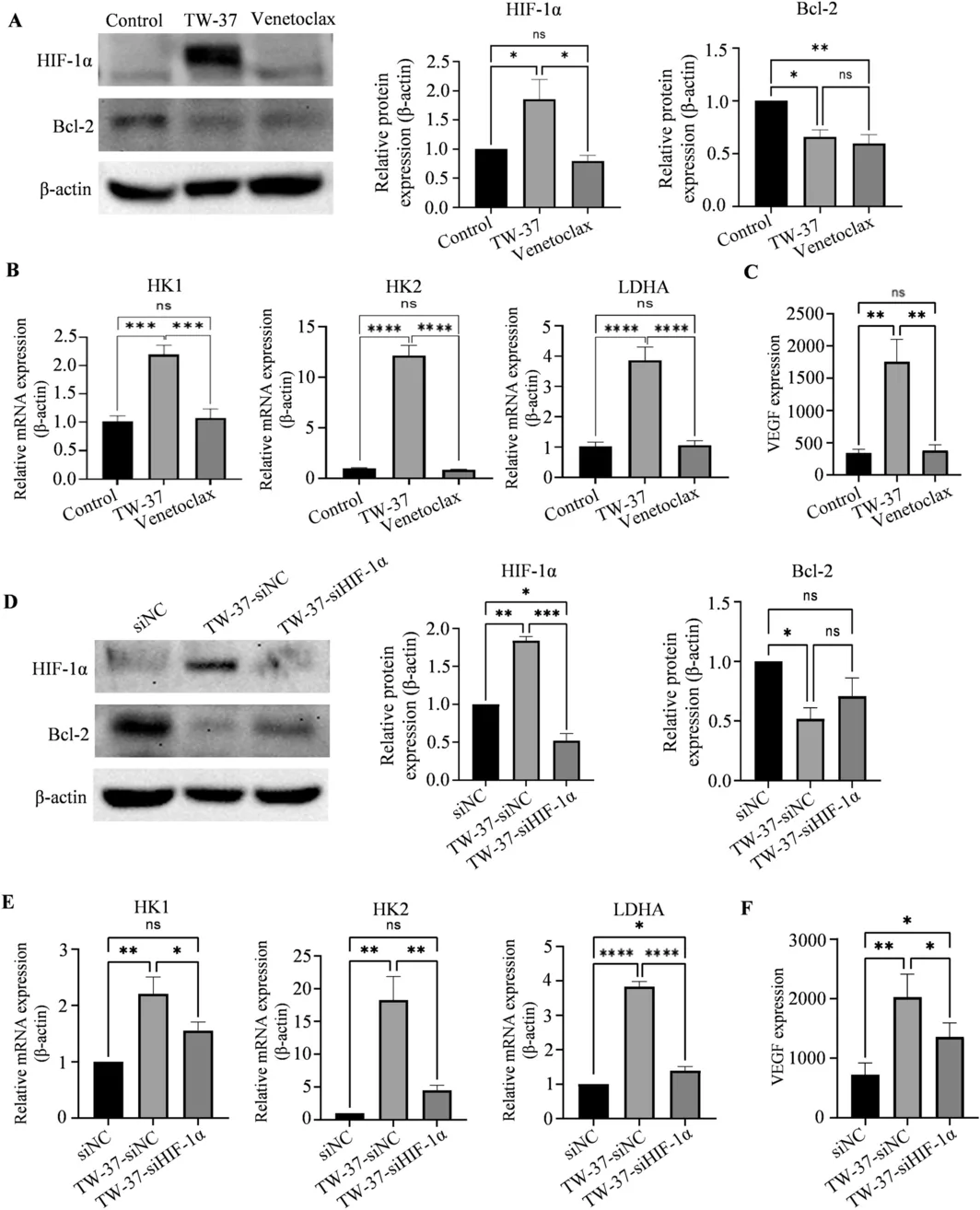
TW‐37‐mediated angiogenic potential is HIF‐1α‐dependent and independent of Bcl‐2 inhibition. (A) Representative western blot images showing protein levels of HIF‐1α and Bcl‐2 in SHED following treatment with TW‐37 or venetoclax; β‐actin served as the loading control. (B) mRNA expression levels of HIF‐1α downstream glycolytic markers *HK1*, *HK2*, and *LDHA* following treatment, analysed by qPCR. (C) Secreted VEGF levels in conditioned media measured by ELISA. (D–F) Impact of HIF‐1α silencing (siHIF‐1α) versus control (siNC) on TW‐37‐treated SHED, showing (D) Western blot of HIF‐1α stabilization and Bcl‐2 inhibition, (E) qPCR analysis of HIF‐1α downstream glycolytic gene expression, and (F) VEGF secretion. Data are presented as mean ± SD of three independent experiments. *n* = 3, *p < 0.05, **p < 0.01, ***p < 0.001, ****p < 0.0001 compared to the control.

As TW‐37 was originally designed as an inhibitor of Bcl‐2, we investigated whether the pro‐angiogenic effects of TW‐37 were a consequence of Bcl‐2 inhibition by comparing its activity with that of venetoclax, a highly selective Bcl‐2 inhibitor. When SHED were treated with 10 μM TW‐37 or 1 μM venetoclax for 24 h, western blot analysis revealed that while both TW‐37 and venetoclax successfully downregulated Bcl‐2 protein expression, only TW‐37 induced significant stabilization of HIF‐1α (Figure 7A). Correspondingly, TW‐37 treatment significantly upregulated the mRNA levels of HIF‐1α downstream targets *HK1*, *HK2*, and *LDHA* (Figure 7B), and increased VEGF secretion as measured by ELISA (Figure 7C). In contrast, venetoclax treatment failed to induce any changes in HIF‐1α protein levels or its downstream transcriptional targets, demonstrating that Bcl‐2 inhibition alone does not account for the hypoxia‐mimetic activity of TW‐37.

To establish the causal role of HIF‐1α, we performed genetic knockdown using siRNA. In SHED transfected with control siRNA, TW‐37 treatment effectively stabilized HIF‐1α and induced the expression of *HK1*, *HK2*, *LDHA*, and VEGF (Figure 7D–F). However, in HIF‐1α‐silenced cells, TW‐37 failed to induce HIF‐1α stabilization or activate these downstream pro‐angiogenic and glycolytic markers. These results definitively demonstrated that TW‐37‐mediated angiogenic potential in SHED is fundamentally dependent on the stabilization of HIF‐1α, rather than merely secondary to Bcl‐2 inhibition and off‐target stress.

### 3.8 Transient Cytostatic Effects of TW‐37 and Recovery of Proliferative Capacity Following Washout

To evaluate the safety and translational feasibility of our preconditioning protocol, we performed a TW‐37 washout experiment to monitor cellular recovery and sustained functional changes. SHED were treated with 10 μM TW‐37 for 24 h, followed by the removal of the compound and cultivation in standard growth media for 7 days. CCK‐8 assays revealed a transient reduction in total metabolic activity on days 1, 3, and 5 post‐washout compared to untreated controls (Figure S4). However, the preconditioned SHED exhibited a “catch‐up” growth phase, with cell viability and numbers returning to control levels by day 7. This pattern suggests that TW‐37 induces a temporary, cytostatic adaptation—likely a period of metabolic and transcriptional reprogramming toward HIF‐1α stabilization—rather than irreversible cytotoxicity. Importantly, despite the removal of the compound, VEGF expression remained significantly elevated, indicating that a 24‐h exposure to TW‐37 is sufficient to “reprogramme” SHED into a pro‐angiogenic state, providing a clinically relevant window for therapeutic effect without the need for continuous drug exposure.

These in vitro findings were further validated by in vivo assessments of SHED‐laden Matrigel plugs (Figure S5). TUNEL staining at 3 days post‐implantation showed no significant difference in apoptosis between TW‐37‐preconditioned cells and controls. Conversely, Ki67 staining demonstrated a significant increase in the proliferation of the preconditioned SHED in the in vivo environment. Together, these results suggest that the TW‐37 preconditioning stimulus is transient and appears well‐tolerated within the observed time‐frame, inducing a pro‐angiogenic phenotype that persists after the chemical trigger is removed. This pattern provides preliminary evidence for the reversibility of our preconditioning strategy, representing a promising step toward meeting the requirements for translational applications.

## 4 Discussion

HIF‐1α stabilization has been proven to enhance the angio−/vasculogenic properties of SHED and boost the pulp regeneration by SHED (Han et al. 2020, 2022). CoCl_2_ and DFO are two commonly used hypoxia mimetic agents that mimic hypoxic conditions by stabilizing HIF‐1α (Jiang et al. 2014; Taheem et al. 2018) and are widely applied in dental cells, such as dental pulp cells, periodontal ligament cells, and gingival fibroblasts (Agis et al. 2012; Du et al. 2021; Müller et al. 2012; Osathanon et al. 2015). However, both CoCl_2_ and DFO exhibit remarkable toxicity to the cells at the effective concentrations. Furthermore, CoCl_2_ and DFO may not reliably mimic the physiological response to hypoxia with respect to targets downstream of HIF‐1α stabilization (Borcar et al. 2013). In addition to being a hypoxia mimetic agent, DFO is a Food and Drug Administration (FDA)‐approved iron chelator used to address systemic iron excess in disorders such as thalassaemia and sickle cell disease (Hatcher et al. 2009). The downside is that its effectiveness is constrained by its nonspecific toxicity at high doses, a short half‐life that affects patient compliance, and the potential to cross the blood–brain barrier (Farr and Xiong 2021). In this study, CoCl_2_ and DFO were first used to examine their function as hypoxia mimetic agents on the expression of HIF‐1α and VEGF in SHED. It was suggested that CoCl_2_ and DFO could also induce HIF‐1α accumulation and promote VEGF expression in SHED. However, they showed significant cytotoxicity at the concentrations that could remarkably activate HIF‐1α and elevate VEGF. Therefore, new hypoxia mimetic agents were explored.

This study identified three new hypoxia mimetic agents, TW‐37, ML228, and CPX, that stabilized HIF‐1α and increased VEGF secretion in a dose‐dependent manner in SHED. While our study primarily focuses on the HIF‐1α‐VEGF axis as a central driver of SHED‐mediated angiogenesis, we acknowledge that neovascularization is a complex, multi‐factorial process involving various pro‐angiogenic signalling molecules. Other factors, such as Angiopoietin‐1 (Ang‐1), basic Fibroblast Growth Factor (bFGF), and Placental Growth Factor (PlGF) (Carmeliet and Jain 2011), also contribute to vessel maturation and stabilization. Given our previous findings identifying VEGF as the dominant effector in HIF‐1α‐stabilized‐SHED‐induced angiogenic responses (Han et al. 2020; Han et al. 2021), we prioritized this master regulator for detailed mechanistic investigation. However, it is important to acknowledge that HIF‐1α stabilization orchestrates a complex secretome. Other factors downstream of HIF‐1α, such as angiopoietins and glycolytic enzymes, likely contribute to the integrated pro‐angiogenic and metabolic shift in SHED.

To ensure the robustness of our conclusions beyond molecular expression, we complemented our VEGF data with functional assays, including in vitro tube formation and in vivo Matrigel plug perfusion. These functional outcomes confirm that the observed VEGF induction translates into a definitive pro‐angiogenic phenotype, though future studies may further explore the synergistic roles of auxiliary growth factors in this model. In this study, TW‐37 was identified as a novel HIF‐1α stabilizer. TW‐37 is a small‐molecule inhibitor of Bcl‐2, designed using a structure‐based approach, with anti‐cancer activity and potential as an apoptosis‐inducing agent for cancer treatment (Ahn et al. 2019; Mori et al. 2021; Yang et al. 2020). Although ML228 (Theriault et al. 2012) and CPX (Linden et al. 2003) have demonstrated established HIF‐1α‐stabilizing effects and VEGF upregulation, neither of which has been investigated in relation to dental stem cells such as SHED. But a study showed that ML228 could increase the expression level of HIF‐1α and collagen‐I while simultaneously inhibiting the cell viability in human gingival fibroblasts (Lifshits et al. 2023). In addition, CPX is an antifungal agent approved by the FDA and also has anti‐cancer effects (Feng et al. 2020; Qi et al. 2020). In this study, we identified that TW‐37, ML228, and CPX were new HIF‐1α stabilizers functioning as hypoxia mimetic agents in SHED, with significant potential for angiogenesis and tissue regeneration.

The three compounds inhibited SHED cellular viability, especially with long‐term treatment at high concentrations. The compounds with high concentrations (20 μM TW‐37, 1/2.5/5 μM ML228, and 10 μM CPX) suppressed the cell viability at 24 h, while almost no dead cells were observed by the live/dead assay, which indicated that the three drugs negatively regulated the cell growth without inducing cellular death in short‐term treatment. A study showed that TW37 exhibits an IC_50_ of 1.8 μM for endothelial cells but demonstrates no cytotoxicity toward fibroblasts at concentrations up to 50 μM (Zeitlin et al. 2006), which suggests that SMs may have various functions in different types of cells and culture conditions. Interestingly, after treatment with the SMs for 24 h, respectively, although there was significant cytotoxicity to SHED at the concentrations of 20 μM (TW‐37), 5 μM (ML228), and 10 μM (CPX), HIF‐1α had the highest expression at those concentrations in each group. It may suggest that neither molecular cytotoxicity nor SHED's inability to proliferate at concentrations above a certain level inhibits HIF‐1α activation at 24 h. Moreover, it was observed that the compounds caused elongated changes in the cell morphology of SHED. This alteration may be due to the changes in the cytoskeletal dynamics and the modulation of cellular signalling pathways, as it has been proven that some SMs targeting actin, microtubules, or focal adhesions could induce cell shape changes (Fenteany and Zhu 2003). In addition, the inhibition of cell proliferation may provide more space for the cells to expand.

Studies have proven that the viability and regenerative capabilities of the transplanted bone marrow mesenchymal stem cells were markedly improved with hypoxic preconditioning (Wei et al. 2012). In cardiac progenitor cells, hypoxic preconditioning enhanced survival rates by suppressing the PI3K/AKT‐DNMT1‐p53 signalling pathway (Xu et al. 2016). Those findings demonstrated that cells preconditioned in a hypoxic microenvironment may exhibit increased cell survival rates and elevated proangiogenic potential. Therefore, in this study, the three SMs were applied in the hypoxic preconditioning of SHED to examine the VEGF secretion and angiogenic property. It has been known that VEGF is recognized as a key angiogenic target gene due to its direct activation by HIF‐1α and its essential function in HIF‐1α‐mediated neovascularization (Oladipupo et al. 2011). The enhanced VEGF signalling could significantly promote the endothelial sprouting (Kugeratski et al. 2019). The increased vascular‐like tube formation and vascular sprouting by HUVECs cultured with the CM from the preconditioned SHED with TW‐37, ML228, and CPX demonstrated the paracrine effects of SHED on angiogenesis by upregulating the VEGF secretion. As dental‐derived mesenchymal stem cells have the capability to function as angioblast‐like cells (Zhang et al. 2016) and HIF‐1α‐stabilization by PHD2 knockdown in SHED alone can promote the in vivo angio−/vasculogenesis without HUVECs (Han et al. 2020), in this study, SHED were pretreated with TW‐37, ML228, and CPX for 24 h and implanted with Matrigel mixture in vivo to examine the vascular property of SHED. The results suggested that the SMs enhanced the angiogenic capability of SHED in vivo. It is reported that VEGF could induce the migration and proliferation of endothelial cells (Jia et al. 2016) and is involved in the rapid mobilization of endothelial progenitor cells in ischemic rat models (Fadini et al. 2006). In our previous study, PHD2 knockdown SHED accelerated the migration and recruitment of HUVECs and had more host‐originated vessels in the Matrigel plugs, indicating a higher vascularization ability in vivo (Han et al. 2020). In accordance with those results, this study found a higher number of perfused vessels and mouse CD31‐positive SHED were observed in the pretreated SHED plugs compared with the non‐treated ones due to the elevation of VEGF expression by the hypoxic preconditioning of SHED with the hypoxia mimetic agents. Additionally, the high expression of human CD31 in the pretreated cells may also be a result of the upregulated VEGF, as VEGF has been proven to induce the endothelial differentiation of SHED (Han et al. 2020). However, not all the cells in the Matrigel plugs expressed CD31, which may be ascribed to the fact that only a unique sub‐population of SHED expressed high VEGFR1 (Bergamo et al. 2021) and a fraction of SHED exhibited endothelial cell characteristics, without any induction (Han et al. 2020). Therefore, the pretreated SHED with the three compounds may act as angioblasts and give rise to the vascular structures, which could mimic the PHD2 knockdown SHED in our previous study (Han et al. 2020).

In this study, 10 μM TW‐37, 1 μM ML228, and 7 μM CPX had varying degrees of effect on VEGF expression and cell viability in vitro. Both 10 μM TW‐37 and 7 μM CPX could significantly elevate the secretory protein levels of VEGF at 24 h without inducing cytotoxicity in SHED. Although 1 μM ML228 inhibited cell viability at 24 h, it induced a higher level of VEGF in SHED than 0.5 μM ML228, and there was no remarkable difference in cell proliferation between the two concentrations. 10 μM TW‐37 had a stronger ability to promote the secretion of VEGF than 1 μM ML228 and 7 μM CPX. Meanwhile, when comparing the cytotoxicity of the three SMs on SHED at the above concentrations, it was indicated that with the treatment of TW‐37 and CPX for 24 h, the cell viability exceeded 80% whereas it was lower than 80% in ML228. According to ISO 10993‐5, percentages of cell viability beyond 80% were considered as non‐cytotoxicity, 80%–60% weak, 60%–40% moderate and below 40% reflect strong cytotoxicity (ISO 10993‐5 2009), indicating acceptable biocompatibility for further therapeutic exploration of TW‐37 and CPX with the cell viability percentages above 80% at 24 h. Obviously, 10 μM TW‐37 exhibited more pronounced effects on both the secretion of VEGF and cell viability in SHED compared to 1 μM ML228 and 7 μM CPX at 24 h. Additionally, TW‐37 had a better effect than ML228 and CPX on in vitro tube formation and sprouting by HUVECs, which may be due to its higher induction of VEGF. More interestingly, preconditioning of SHED by TW‐37 at a concentration of 10 μM, ML228 at 1 μM, and CPX at 7 μM for 24 h was applied in the in vivo Matrigel plug implants, in which TW‐37‐preconditioned SHED showed the strongest angiogenic effect. It follows that although all three drugs stimulated HIF‐1α and VEGF expression, they varied in their efficacy and ability to promote the expression and influence the functionality at the applied concentrations. Obviously, in this study, TW‐37 exhibited the optimal functionality on the angiogenesis of SHED in vitro and in vivo.

Both ML228 and CPX have published evidence confirming their angiogenic effects are HIF‐1α‐dependent (Theriault et al. 2012; Linden et al. 2003). ML228 was identified as a direct small‐molecule HIF pathway activator via high‐throughput HRE reporter assays and stabilizes HIF‐1α protein, promotes nuclear translocation, and induces expression of the canonical HIF‐1α target gene VEGF (Theriault et al. 2012). Mechanistically, ML228 acts as an iron chelator, inhibiting HIF‐1α prolyl hydroxylation and degradation (Theriault et al. 2012). CPX is a lipophilic iron chelator that stabilizes HIF‐1α under normoxia by inhibiting prolyl hydroxylases and activates HIF‐1‐mediated reporter gene activity and upregulates VEGF mRNA and protein (Linden et al. 2003). In the chick chorioallantoic membrane assay, CPX was proven to directly induce angiogenesis, which was abrogated by HIF‐1α inhibition (Linden et al. 2003). In this study, it also showed that the pro‐angiogenic effects of TW‐37 in SHED were HIF‐1α–dependent.

RNA‐sequencing analysis revealed a significant differential gene expression pattern in SHED under the stimulation of TW‐37 compared to the well‐known hypoxia mimetic agent, CoCl_2_. The pathway enrichment analysis revealed that the pathways enriched were involved in hypoxia, cell survival, cell proliferation, and cellular metabolism. The observations on pathway enrichment may give a clue that TW‐37‐treated SHED had a superior angiogenic property than CoCl_2_‐treated SHED, and the mechanism of action of TW‐37 on SHED might be distinct from the currently recognized PHD inhibitors, such as CoCl_2_.

However, a limitation of the present study is the reliance on a single, commercially sourced SHED population. While this approach minimized experimental noise and allowed for a clear mechanistic characterization of the three small molecules, it is well‐established that mesenchymal stem cells exhibit significant donor‐to‐donor variability in terms of proliferation and secretory profile (Kukaj et al. 2025; Lyu et al. 2024). Consequently, the optimal dosing and the magnitude of VEGF induction observed here may vary across different cell lots or biological donors, and therefore, the optimal dosing regimens identified here should be viewed as a baseline. Future studies incorporating multiple independent SHED donors will be essential to confirm the robustness of these preconditioning strategies and to ensure the generalizability of our findings for broader clinical applications.

In addition, a critical safety concern is the potential carryover of the residual preconditioning components in the final cell products (Chialastri et al. 2024). While TW‐37 exhibits dose‐dependent effects on cell viability, our washout studies demonstrated that this impact is transient and reversible. The observed recovery of proliferative capacity by Day 7, coupled with a lack of increased apoptosis in vivo, suggests that the preconditioning protocol triggers a survival‐oriented metabolic shift rather than irreversible damage. However, while these results are encouraging for short‐term preconditioning, the safety profile remains preliminary. Further long‐term studies are required to rule out delayed effects on genomic stability or lineage‐specific differentiation before clinical implementation. From a regulatory perspective, this ‘hit‐and‐run’ approach is advantageous; by removing the compound prior to transplantation, we minimize the risk of residual systemic toxicity. Cell products exposed to small‐molecule preconditioning agents may be classified as combination products or at least require additional scrutiny regarding their manufacturing process (Dou et al. 2024). The manufacturing under current good manufacturing practices may necessitate rigorous quality control, including batch‐to‐batch consistency of the preconditioning itself, demonstration of effective compound removal, and stability testing of the final cell product (Fury et al. 2025). Therefore, while this proof‐of‐concept study establishes the biological rationale for hypoxia‐mimetic preconditioning, future clinical translation will require rigorous pharmacodynamic studies to satisfy FDA or EMA requirements for chemically modified advanced therapy medicinal products (ATMPs).

## 5 Conclusions

This proof‐of‐concept study identified three small‐molecule hypoxia mimetic agents, TW‐37, ML228, and CPX, that effectively stabilize HIF‐1α expression in SHED and enhance the pro‐angiogenic properties in a dose‐dependent manner. Preconditioning with 10 μM TW‐37, 1 μM ML228, or 7 μM CPX for 24 h promotes robust angiogenic activity in vitro and in vivo, largely driven by increased VEGF secretion alongside other HIF‐1α‐regulated metabolic adaptations. Notably, we report for the first time that TW‐37 acts as a potent hypoxia mimetic, demonstrating superior HIF‐1α stabilization and angiogenic enhancement compared with conventional agents. Mechanistic validation confirmed that these effects are specifically mediated through a direct HIF‐1α‐dependent pathway, independent of Bcl‐2 inhibition. Furthermore, washout experiments and short‐term in vivo assessments suggest that this preconditioning triggers a reversible, cytostatic adaptation rather than terminal cytotoxicity. While these findings support the translational potential of “hit‐and‐run” small‐molecule preconditioning in regenerative endodontics, further studies are required to confirm these optimal dosages across diverse donor populations and to evaluate long‐term safety beyond the current observation window.

## Author Contributions

**Hong Wang:** study design, data acquisition, data analysis, visualization, writing – original draft, review and editing. **Jiawei Liu and Xintao Yang:** data acquisition, data analysis, writing – review and editing. **Mohamad Koohi‐Moghadam:** study design, data analysis, writing – review and editing. **Shuting Gao, Yuanyuan Han, and Dineshi Sewvandi Thalakiriyawa:** data analysis, writing – review and editing. **James Kit Hon Tsoi and Yanqi Yang:** writing – review and editing. **Waruna Lakmal Dissanayaka:** conceptualization, study design, supervision, project administration, funding acquisition, writing – review and editing.

## Funding

This work was supported by the General Research Fund (Project Number GRF17125421), Research Grants Council, Hong Kong.

## Conflicts of Interest

The authors declare no conflicts of interest.

## Supporting information


**Figure S1:** Preferred reporting items for laboratory studies in endodontology (PRILE) 2021 flowchart.
**Figure S2:** Representative micrographs and quantification of Live/Dead assays for SHED treated with TW‐37, ML228, and CPX at 48 and 72 h. SHED treated with TW‐37 for (A) 48 h and (B) 72 h. SHED treated with ML228 for (C) 48 h and (D) 72 h. SHED treated with CPX for (E) 48 h and (F) 72 h. Positive control: SHED treated with 70% ethanol as a reference for dead cells (far right column). Scale bar = 500 μm. *n* = 3, *p < 0.05, **p < 0.01, and ***p < 0.001.
**Figure S3:** Inverted light microscopy images showing the cell morphology of SHED following treatment with (A) TW‐37, (B) ML228, and (C) CPX for 24 h. Scale bar = 500 μm.
**Figure S4:** Recovery of cell metabolic viability following TW‐37 washout. SHED were preconditioned with 10 μM TW‐37 for 24 h, followed by washout and cultivation in standard growth media. Cell metabolic activity was assessed via CCK‐8 assay at days 1, 3, 5, and 7 post‐washout. Data show a transient decrease in metabolic activity followed by a recovery to control levels by day 7, indicating a reversible cytostatic effect. Data are presented as mean ± SD (*n* = 3). ***p < 0.001compared to control.
**Figure S5:** In vivo safety and proliferation of TW‐37‐preconditioned SHED. (A) Representative images and quantitative analysis of TUNEL staining in Matrigel plugs at 3 days post‐implantation, showing no significant difference in apoptotic cell percentage between control and TW‐37‐pretreated groups. (B) Representative images and quantitative analysis of Ki67 immunofluorescence staining, demonstrating significantly enhanced proliferation in the TW‐37‐preconditioned SHED in vivo. Data are presented as mean ± SD (*n* = 3). ***p < 0.001compared to the control.
**Table S1:** Summary of RNA Sequencing Data Quality Control.
**Table S2:** Gene sets related to hypoxia, angiogenesis, glycolysis, odontogenesis, oxidative phosphorylation, and cell survival as identified from the GeneCards database.

## Data Availability

The data that support the findings of this study are available on request from the corresponding author. The data are not publicly available due to privacy or ethical restrictions.
