# Supplementary material for: Optimal Hypoxia Mimetic Small Molecules for Enhancing Angiogenic Properties of Stem Cells From Human Exfoliated Deciduous Teeth
Source: Int Endod J. 2026 Jun 2;59(10):2122–42. doi: 10.1111/iej.70191 (PMC13569154; doi:10.1111/iej.70191)
Supplement: Supplementary file 1 [file IEJ-59-2122-s001.docx]

RATIONALE/JUSTIFICATION Current agents that function as HIF-1α stabilizers are associated with low efficacy and high cytotoxicity in relation to SHED.

AIM/HYPOTHESIS To identify an optimal small molecule HIF-1α stabilizer for SHED and examine its angiogenic effects in vivo

ETHICAL APPROVAL The Committee on the Use of Live Animals in Teaching and Research (CULATR, Reference No. 24-218) of the University of Hong Kong

SAMPLES In vivo Matrigel plugs; 6 plugs per group

EXPERIMENTAL AND CONTROL GROUPS, INCLUDE INDEPENDENT VARIABLES Control group, TW-37 group, ML228 group, CPX group, n=6; 2 Matrigel plugs per mouse

OUTCOME(S) ASSESSED, INCLUDE DEPENDENT VARIABLES AND TYPE Blood vessel formation, mouse CD31, and human CD31

METHOD USED TO ASSESS THE OUTCOME (S) AND WHO ASSESSED THE OUTCOME(S) H&E staining and IHC staining. All outcomes were assessed by the authors of this study.

RESULTS SHED pretreated with the small molecules (10 μM TW-37, 1 μM ML228, and 7 μM CPX) for 24 h in vitro showed a significant increase in the vascularization after in vivo implantation.

CONCLUSION(S ) Hypoxic preconditioning with TW-37, ML228, and CPX pre-treatment boosted the angio-/vasculogenesis of SHED in vivo.

FUNDING DETAILS This study was supported by General Research Fund (Project Number-17125421), Research Grants Council, Hong Kong

CONFLICT OF INTEREST None

**Figure S1.** Preferred Reporting Items for Laboratory studies in Endodontology (PRILE) 2021 flowchart.

**Figure S2.** Representative micrographs and quantification of Live/Dead assays for SHED treated with TW-37, ML228, and CPX at 48 and 72 hours. SHED treated with TW-37 for (**A**) 48 h and (**B**) 72 h. SHED treated with ML228 for (**C**) 48 h and (**D**) 72 h. SHED treated with CPX for (**E**) 48 h and (**F**) 72 h. Positive control: SHED treated with 70% ethanol as a reference for dead cells (far right column). Scale bar = 500 μm. n=3, **P*<0.05, ***P*<0.01, and ****P*<0.001.

**Figure S3**. Inverted light microscopy images showing the cell morphology of SHED following treatment of (**A**) TW-37, (**B**) ML228, and (**C**) CPX for 24 h. Scale bar = 500 μm.

**Figure S4.** Recovery of cell metabolic viability following TW-37 washout.

SHED were preconditioned with 10 μM TW-37 for 24 hours, followed by washout and cultivation in standard growth media. Cell metabolic activity was assessed via CCK-8 assay at days 1, 3, 5, and 7 post-washout. Data show a transient decrease in metabolic activity followed by a recovery to control levels by day 7, indicating a reversible cytostatic effect. Data are presented as mean ± SD (n=3). ****P*<0.001compared to control.

**Figure S5**. In vivo safety and proliferation of TW-37-preconditioned SHED.

(**A**) Representative images and quantitative analysis of TUNEL staining in Matrigel plugs at 3 days post-implantation, showing no significant difference in apoptotic cell percentage between control and TW-37-pretreated groups. (**B**) Representative images and quantitative analysis of Ki67 immunofluorescence staining, demonstrating significantly enhanced proliferation in the TW-37-preconditioned SHED in vivo. Data are presented as mean ± SD (n=3). ****P*<0.001compared to the control.

**Supplementary Table 1:** Summary of RNA Sequencing Data Quality Control.

| **Samples** | **Clean reads** | **Clean bases** | **GC content (%)** | **Q30 (%)** |
| --- | --- | --- | --- | --- |
| CoCl_2_-24h-1 | 23126267 | 6920410424 | 49.16 | 93.84 |
| CoCl_2_-24h-2 | 26232599 | 7848082806 | 49.29 | 93.85 |
| CoCl_2_-24h-3 | 22280322 | 6667741410 | 49.19 | 93.88 |
| CoCl_2_-3h-1 | 23621244 | 7066659490 | 48.58 | 94.44 |
| CoCl_2_-3h-2 | 29783567 | 8910712436 | 48.81 | 94.2 |
| CoCl_2_-3h-3 | 22445393 | 6714320962 | 48.32 | 94.78 |
| TW-37-24h-1 | 23101602 | 6912326206 | 48.63 | 93.86 |
| TW-37-24h-2 | 29996184 | 8976258152 | 48.87 | 94.12 |
| TW-37-24h-3 | 23966014 | 7171458890 | 48.69 | 93.63 |
| TW-37-3h-1 | 22553768 | 6749979000 | 48.87 | 94.02 |
| TW-37-3h-2 | 22989739 | 6879043698 | 49.08 | 93.81 |
| TW-37-3h-3 | 23821293 | 7129302396 | 48.92 | 94.52 |

Clean reads: Counts of clean PE reads; Clean bases: total base number of Clean Data; GC content (%): Percentage of G, C in clean data; Q30 (%): Percentage of bases with Q-score no less than Q30.

**Supplementary Table 2:** Gene sets related to hypoxia, angiogenesis, glycolysis, odontogenesis, oxidative phosphorylation, and cell survival as identified from the GeneCards database.

| **Angiogenesis-related genes** | **Hypoxia-related genes** | **Glycolysis-related genes** | **Odontogenesis-related genes** | **Oxidative phosphorylation-related genes** | **Cell survival-related genes** |
| --- | --- | --- | --- | --- | --- |
| VEGFA | ADM | ALDOA | BMP2 | AIFM1 | CCND1 |
| PDGFB | DDIT4 | ENO2 | KRT14 | FARS2 | EGFR |
| TGFB2 | PGF | HK2 | PHLDB3 | GFM1 | FAS |
| NOTCH1 | EGLN3 | LDHA | TGFB1 | GFM2 | NOTCH1 |
| FLT1 | SERPINE1 | PFKFB3 | ZNF222 | PNPT1 | CASP3 |
| FGFR1 | CA9 | PFKP | SERPINE1 | TARS2 | MYC |
| EFNB2 | HILPDA | PGAM1 | FOS | MAPK14 | HIF1A |
| PGF | EGLN1 | PGK1 | TGIF1 | CAT | PIK3R1 |
| FGF2 | PTGS2 | SLC2A1 | EGR1 | SOD1 | PTCH1 |
| EGFR | BNIP3 | TPI1 | TUFT1 | POLRMT | CDKN2A |
| ANG | NDRG1 | GPI | DSPP | ALDH3A2 | ERBB2 |
| CXCL8 | PDK1 | RRAD | OCN | CDK1 | KIT |
| ETS1 | SLC2A1 | TIGAR | MPC1 | SOD2 | BCL2 |
| IL6 | HMOX1 | ENO1 | PAX9 | MRPS34 | MAPK1 |
| PTGS2 | VEGFA | ENO3 | STAT3 | NDUFS7 | MTOR |
